# Objective preclinical measures for bone conduction implants

**DOI:** 10.3389/fnins.2024.1324971

**Published:** 2024-03-14

**Authors:** Irina Wils, Alexander Geerardyn, Tristan Putzeys, Guy Fierens, Kathleen Denis, Nicolas Verhaert

**Affiliations:** ^1^Department of Neurosciences, Experimental Otorhinolaryngology, KU Leuven, Leuven, Belgium; ^2^Department of Otorhinolaryngology, Head and Neck Surgery, University Hospitals Leuven, Leuven, Belgium; ^3^Laboratory for Soft Matter and Biophysics, Department of Physics and Astronomy, KU Leuven, Leuven, Belgium; ^4^Cochlear Technology Centre Belgium, Mechelen, Belgium; ^5^Biomechanics Section, Department of Mechanical Engineering, KU Leuven, Leuven, Belgium

**Keywords:** bone conduction, intracochlear pressure, promontory velocity, ear canal pressure, objective measures

## Abstract

The study evaluates the accuracy of predicting intracochlear pressure during bone conduction stimulation using promontory velocity and ear canal pressure, as less invasive alternatives to intracochlear pressure. Stimulating with a percutaneous bone conduction device implanted in six human cadaveric ears, measurements were taken across various intensities, frequencies, and stimulation positions. Results indicate that intracochlear pressure linearly correlates with ear canal pressure (*R*^2^ = 0.43, RMSE = 6.85 dB), and promontory velocity (*R*^2^ = 0.47, RMSE = 6.60 dB). Normalizing data to mitigate the influence of stimulation position leads to a substantial improvement in these correlations. *R*^2^ values increased substantially to 0.93 for both the ear canal pressure and the promontory velocity, with RMSE reduced considerably to 2.02 (for ear canal pressure) and 1.94 dB (for promontory velocity). Conclusively, both ear canal pressure and promontory velocity showed potential in predicting intracochlear pressure and the prediction accuracy notably enhanced when accounting for stimulation position. Ultimately, these findings advocate for the continued use of intracochlear pressure measurements to evaluate future bone conduction devices and illuminate the role of stimulation position in influencing the dynamics of bone conduction pathways.

## 1 Introduction

Bone conduction implants offer a potential solution for patients with conductive, mixed, and even sensorineural hearing loss (https://www.who.int/publications/i/item/9789240020481). However, the invasive nature of these implants, either percutaneous, active, or passive transcutaneous, necessitates preclinical testing on cadaveric specimens before conducting patient trials (ASTM, 2022). In contrast with live patients who can provide subjective feedback to evaluate the hearing sensation, tests in cadaveric specimens require objective measures. Currently, three measures are commonly employed: intracochlear pressure (Greene et al., 2015; Borgers et al., 2019; Mattingly et al., 2020; Fierens et al., 2022; Putzeys et al., 2022), promontory velocity (Stenfelt and Goode, 2005; Rosowski et al., 2007; Dobrev et al., 2016, 2020, 2023; Roosli et al., 2016; Rigato et al., 2019; Ghoncheh et al., 2021; Prodanovic and Stenfelt, 2021), and ear canal pressure (Mertens et al., 2014; Nie et al., 2022).

The process of perceiving sound through air conduction follows a clear trajectory: sound waves enter the ear canal, induce vibrations in the tympanic membrane, are amplified by the ossicles, and are transmitted to the cochlea. In contrast, the perception of sound through bone conduction remains mostly theoretic, with uncertainties regarding the significance and relative contributions of these pathways (Tsai et al., 2005; Chhan et al., 2013). Objectively measuring the velocity of specific structures and the pressure within these structures can help resolve these uncertainties.

Intracochlear pressure measurements involve inserting one or two miniature pressure sensors in the cochlear spaces filled with inner ear fluid, specifically in the scala vestibuli and the scala tympani. The differential pressure - i.e., the complex difference between the pressure in both scalae - correlates with the cochlear drive and can thus be used to estimate hearing sensation (Dancer and Franke, 1980; Niesten et al., 2015; Borgers et al., 2018; Stieger et al., 2018; Guan et al., 2020). One advantage of this measurement technique is that it encompasses the contributions of all five bone conduction pathways: ear canal compression, middle ear inertia, inner ear compression, inner ear inertia, and transmission via the cerebrospinal fluid (Stenfelt and Goode, 2005). However, it should be noted that this technique is highly invasive and requires a canal wall-up mastoidectomy, an enlarged posterior tympanotomy, and the removal of the mastoidal portion of the facial nerve to gain access to the cochlea. Additionally, the cochlea must be surgically opened to insert the sensors. Another limitation of this technique is the relatively low signal-to-noise ratio, which necessitates either high-level stimulation of the hearing implant (up to 100 dB HL) (Creighton et al., 2016; Borgers et al., 2019) or extensive filtering and prolonged measurement times (Putzeys et al., 2022).

Promontory velocity can be measured with laser Doppler vibrometry, a non-contact optical measurement technique. It is the current standard method to evaluate middle ear implants (ASTM, 2022). Unlike intracochlear pressure measurements, this technique solely captures the movement of the cochlea, representing only two pathways: inner ear inertia and compression. While it is an invasive procedure, as the cochlear surface must be visible, it does not require the cochlear capsule to be surgically opened. Access can be achieved through a canal wall-up mastoidectomy, an enlarged posterior tympanotomy without facial nerve removal (Rosowski et al., 2007; Dobrev et al., 2020), or through the ear canal when the tympanic membrane is removed (Rigato et al., 2019; Ghoncheh et al., 2021). The main advantage of this technique is its good signal-to-noise ratio.

Ear canal pressure is measured using a probe microphone placed in the ear canal. Although it is less commonly used than intracochlear pressure and promontory velocity measurements for evaluating bone conduction implants (Mertens et al., 2014; Nie et al., 2022), it may still serve as a suitable measure of hearing sensation, considering that ear canal compression is one of the bone conduction pathways. Additionally, the other pathways could contribute to the ear canal pressure through reverse stimulation via the middle ear ossicles (Stenfelt and Goode, 2005). It remains unclear whether this technique accurately predicts hearing sensation. Nevertheless, the technique has a good signal-to-noise ratio and is non-invasive. As a result, it could signify progress in objective measurements for patients, encompassing the perioperative, intraoperative, and postoperative stages. This objective measure would be particularly advantageous for individuals incapable of providing feedback on their hearing sensation, including young children.

In contrast with *ex vivo*, objective, preclinical tests, there also exist several *in vivo*, subjective tests. These psychoacoustical tests evaluate the relationship between an acoustical stimulus or sound and an individual’s perception (Fechner, 1860; Yost, 2015). An example of these tests is a loudness balancing experiment, where normal hearing participants are asked to compare air and bone conduction sounds based on loudness, an experiment that can provide more information about how loud a bone conduction stimulus is perceived (Putzeys et al., 2022).

This study assesses the accuracy, precision, and invasiveness of three objective measures for preclinical testing of bone conduction induced hearing sensation: intracochlear pressure, promontory velocity, and ear canal pressure. The evaluation involves simultaneous measurements using these techniques in various specimens and across different stimulation frequencies, locations, and intensities. Result variations attributable to these factors would indicate that specific measurement techniques can provide more insight into the bone conduction pathways. As those measures are recorded for stimulation at multiple positions, an additional goal is to study which position is the most efficient or generates the highest amplitude for the three objective measures.

## 2 Materials and methods

### 2.1 Specimen preparation

Four fresh-frozen human cadaveric specimens were provided by the Vesalius Institute (Anatomy and Pathology, University of Leuven - KU Leuven, Belgium) following the ethical approval of the same institute (S65502) and following the Helsinki declaration. Similar procedures regarding harvesting and preparation of specimens were used in our previous studies (Borgers et al., 2019; Fierens et al., 2022; Putzeys et al., 2022) and inspired by previous intracochlear pressure measurements (Nakajima et al., 2009; Greene et al., 2015; Grossöhmichen et al., 2016; Stieger et al., 2018; Mattingly et al., 2020).

**Figure 1:**
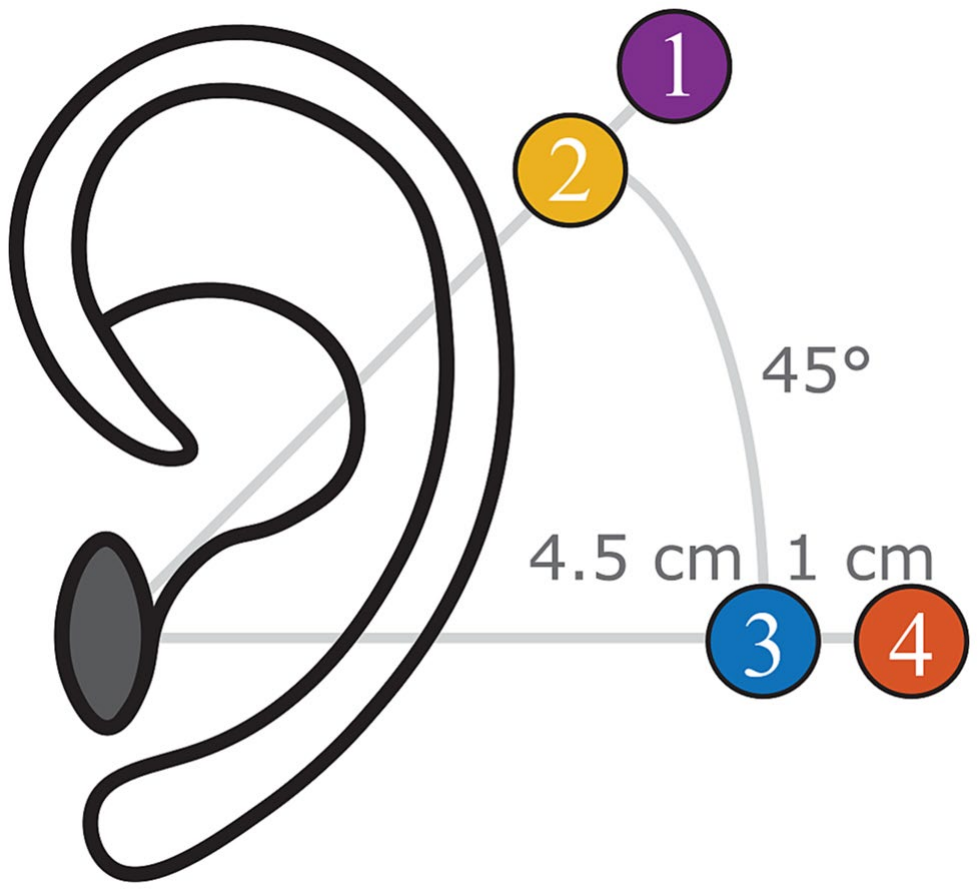
Indication of the four different positions investigated during the experiment (left ear): (1) Standard Baha position, (2) proximal Baha position, (3) standard Osia position, and (4) distal Osia Position.

In brief, all specimens were harvested within 72 h postmortem, following the guidelines described in ASTM-F2504 (American Society for Testing and Materials, 2014). Specimens were frozen at −20°C immediately after harvesting and thawed at 2°C 48–72 h before the experiments. Before starting the experiment, the external ear canal and tympanic membrane were inspected to exclude abnormalities. Thereafter, surgical preparation consisted of a canal wall-up mastoidectomy with enlarged posterior tympanotomy with the removal of the mastoidal portion of the facial nerve. In addition, the cochlear wall was thinned at the level of the scala tympani and the scala vestibuli. Finally, four BI300 bone screws (Cochlear Ltd., Sydney, Australia) were implanted following the manufacturer’s surgical guidelines in the following locations (Figure 1):

1. Standard Baha position: 5.5 cm posterosuperior to the center of the external ear canal at an angle of 45° with respect to the sagittal axis (Cochlear Limited, 2019).
2. Proximal Baha position: 4.5 cm posterosuperior to the center of the external ear canal at an angle of 45° with respect to the sagittal axis.
3. Standard Osia position: 4.5 cm posterior to the center of the external ear canal at an angle of 0° with respect to the sagittal axis (Cochlear Limited, 2019).
4. Distal Osia position: 5.5 cm posterior to the center of the external ear canal at an angle of 0° with respect to the sagittal axis.

During the experiment, a ring of modeling clay (Play-Doh, Hasbro, Pawtucket, USA) was put on a vibration-isolated table (M-VIS3048-SG2-325A, Newport Spectra-Physics, the Netherlands). The head specimen was then placed on its side, with the contralateral side contacting the modeling clay, ensuring that the specimen was not touching the table. During the experiments, the specimens were kept moist with a saline solution.

### 2.2 Stimulation

Stimulation was performed both by air conduction and bone conduction but presented separately to the specimen. Air conduction stimulation was performed via an insert earphone (ER-3C, Etymotic Research, Illinois, USA) in the external ear canal, held in place by an insert phone plug. For bone conduction stimulation, a Baha 5 SuperPower actuator (Cochlear Ltd., Sydney, Australia), i.e., without sound processor electronics, was connected to the bone screws with an abutment at the previously mentioned locations. Signals were generated using Matlab (2018b Mathworks, Massachusetts, USA) and provided to the insert earphone and Baha via a sound card (Fireface UC, RME, Haimhausen, Germany) and an LPA01 amplifier set to a unit gain (Newtons4th Ltd., Leicester, UK) to ensure a constant potential output.

A stepped sine wave ranging from 100 Hz to 10 kHz with 50 logarithmically spaced frequencies at a stimulation voltage of 0.1 V RMS was used to measure the middle and inner ear transfer functions. These transfer functions quantify the ratio of stapes and round window velocities with the ear canal pressure, respectively. The sine sweep was repeated 10 times per measurement.

During the rest of the experiment, stimulation signals lasted 80 s and contained a single-frequency sine signal to stimulate the specimen. Ten different frequencies were used: 250 Hz, 500 Hz, 700 Hz, 800 Hz, 1 kHz, 1.5 kHz, 2 kHz, 3 kHz, 4 kHz, and 6 kHz. The frequency 750 Hz, conventionally used in this type of experiment, was substituted with 700 and 800 Hz due to the presence of a 750 Hz noise source in the measurement chamber. The stimulation amplitude varied across the different frequencies to obtain a sound level of 60, 70, and 80 dB HL. Regarding air conduction, the insert earphones are calibrated with an artificial ear (type 4152, Brüel and Kjaer, Virum, Denmark) according to the ISO 398-2 standard (International Organization for Standardization, 2014).

Regarding bone conduction, the Baha actuator was coupled to a TU-1000 skull simulator (Nobelpharma Inc., Goteborg, Sweden) with a BI300 abutment. For 60 dB HL, the stimulation amplitude was adjusted until the output force level was the same as that from a loudness balancing experiment (Table 1). In this study, healthy participants were asked to match the intensity of the bone conduction stimulus to an air conduction stimulus at 60 dB HL, similar to the loudness balancing study of Putzeys et al. (2022). In the cadaver experiments, the mean of the obtained output force levels is used. The mean for 250 Hz was extrapolated using shape-preserving piecewise cubic extrapolation, while the means for 700 and 800 Hz were interpolated. For stimulation levels 70 and 80 dB HL, the desired output force level was increased by 10 and 20 dB, respectively.

**Table 1:** Output force levels (OFL) from a loudness balancing experiment at 60 dB HL.

Frequency (Hz)	*250	500	*700	750	*800	1000	1500	2000	3000	4000	6000
Mean OFL (dB re 1μN)	105.1	97.5	92.7	91.9	91.3	89.3	68.8	85.2	86.2	90.0	86.3
Standard deviation OFL (dB)	NA	4.0	NA	4.66	NA	2.6	5.1	2.5	5.3	3.8	3.3

*For frequencies 250, 700, and 800 Hz, the mean is obtained with a shape-preserving piecewise cubic extra- and interpolation, and the standard deviation was not applicable (NA). The frequency of 750 Hz was excluded due to a noise source at this frequency in the measurement room.

### 2.3 Measurements

#### 2.3.1 Ear canal pressure

Alongside the insert earphone, a probe tube microphone (ER-7C, Etymotic Research, USA) was mounted in the insert phone plug in the ear canal. A stimulus of 90 dB re 20 μPa at 1 kHz was sent to the insert phone to ensure the correct positioning of the insert phone and probe tube microphone. The microphone then confirms this stimulus. When the microphone readings deviated, this indicated that either the insert phone or microphone was occluded by the ear canal. In that case, the plug was repositioned. The obtained pressure was recorded by an external soundcard (Fireface UC, RME, Haimhausen, Germany).

#### 2.3.2 Laser Doppler vibrometry

A single-point laser Doppler vibrometry system mounted on a surgical microscope (OFV-534 Compact Sensor Head and A-HLV MM 30 Micromanipulator; OFV-5000 Vibrometer controller; Polytec GmbH, Waldbronn, Germany) was used to measure the velocity of the posterior crus of the stapes, the round window membrane, and the promontory. The laser beam was aimed at a 500×500 μm^2^ piece of retroreflective tape (A-RET-T010, Polytec GmbH, Waldbronn, Germany) on those structures, normal to the structure’s area. To enhance visual accessibility to the round window membrane, we removed the overlying bony structure through careful drilling. Subsequently, we applied the reflective tape to the membrane’s center, securing it in position with the help of water surface tension. The output of the laser Doppler vibrometry was recorded by an external soundcard (Fireface UC, RME, Haimhausen, Germany) when the stimulus was a stepped sine wave. However, when the single frequency sine wave was used, data acquisition was performed via a lock-in amplifier (SR830, Stanford Research Systems, Sunnyvale, CA, USA).

#### 2.3.3 Intracochlear pressure

The pressures in both scala vestibuli and tympani were simultaneously measured using two commercial fiber-optic pressure sensors (FOP M-260, FISO Technologies, Canada) with an outer diameter of approximately 310μm. The optical signal of those sensors was converted to an electrical one by an interferometer (OCT Common-Path Interferometer, Thorlabs, Germany) using a low-coherent infrared light source (S5FC1021S - SM Benchtop SLD Source, 1310 nm, 12.5 mW, 85 nm Bandwidth, Thorlabs, Germany). While determining the precise position of the pressure sensor membrane would typically involve signal demodulation, the focus here is on the sine wave’s amplitude. The linearity between the amplitude of the pressure wave and the sensor light intensity facilitated calibration in a water-vibrating column, as previously described by Pfiffner et al. (2017) and Borgers et al. (2019). This calibration established a linear conversion ratio between the sensor-generated potential and the amplitude of the pressure wave. Data was acquired via a lock-in amplifier (SR830, Stanford Research Systems, USA) to overcome the low signal-to-noise ratio.

During sensor insertion, the middle ear was first immersed in saline to prevent air from entering the cochlea. After that, a cochleostomy was drilled in the SV using a diamond burr of 0.5 mm (for blue-lining) and a perforator of 0.35 mm. Then, the sensor was slowly inserted approximately 100 to 300 μm into the scalae using a micromanipulator (MD4, Märzhäuser Wetzlar GMBH & Co.KG, Germany). Similar to the insertion of the insert phone plug, a stimulus of 90 dB re 20 μPa at 1 kHz is sent to the insert phone, and the output of the pressure sensor is checked to ensure that the sensor is not in contact with the cochlear wall. Thereafter, the sensor is sealed using dental impression material (Alginoplast®, Heraeus Kulzer GmBH, Germany). Next, the saline is partially removed, and the sensor is fixed using bone cement (Durelon, 3M, USA) to avoid relative motion between the sensor and the cochlea. After that, a similar procedure is performed for ST. After cementing each sensor, it was released from the micromanipulators to reduce artificial pressure (Borgers et al., 2019).

### 2.4 Experimental procedure and data analysis

#### 2.4.1 Quality control

The middle ear transfer function (METF), i.e., the ratio of the stapes velocity to the ear canal pressure, is measured before and after sensor insertion. Comparing the measured transfer function with the range of Koch et al. (2022) enables us to check whether the specimen is representative of the population. However, this range is not an exclusion criterion. Furthermore, checking whether there is a significant difference between the measurements before and after sensor insertion allows us to check whether mechanical changes occur due to the drilling and insertion procedure. Significance was verified with a paired *t*-test (*α* = 0.05) and a Bonferroni correction for multiple comparisons (*n* = 300, 50 frequencies, and six specimens).

A stepped sine ranging from 100 Hz to 10 kHz with 50 logarithmically spaced frequencies at a stimulation voltage of 0.1 V RMS was presented ten times to the ear canal. The raw signal was divided into synchronized epochs (i.e., time windows). For each epoch, the measured signal was filtered using second-order Butterworth filters with a one-third octave bandwidth that was symmetrical around the center frequencies before calculating the frequency-dependent response.

Additionally, the velocity of the round window was measured equivalent to the stapes velocity. The phase difference between the stapes and round window velocity is expected to be 180° as the cochlear fluid is assumed incompressible, and the round and oval windows are assumed to be the two main fluid outlets (Kringlebotn, 1995; Stenfelt et al., 2004). Thus, this measurement can also be used as a control measurement. Deviations from 180° can indicate a compressible air bubble in the cochlea, the reinforcement of one of the fluid outlets, or the creation of an additional outlet.

#### 2.4.2 Noise measurement

Lock-in amplifiers are effective for removing random noise but not for noise with the same frequency as the measurements. Therefore, a so-called dark (or silent) measurement was performed before the measurements with stimulation. Calculating the complex difference between the recorded values of the stimulation measurements and the dark measurement provides an accurate correction for the noise (Putzeys and Wübbenhorst, 2015; Putzeys et al., 2022).

#### 2.4.3 Air conduction and bone conduction

During these measurements, the insert phone or Baha stimulated the ear with single-frequency sine signals. Both the laser Doppler vibrometer and pressure sensors were read with a lock-in amplifier. Data were processed and analyzed in Matlab (2018b Mathworks, Massachusetts, USA). Processing included converting the recorded potential values, in volts, to pressure in Pa or velocity in mm/s. Next, the differential pressure is calculated as the complex difference between SV and ST pressure. As an individual lock-in amplifier measures the pressure in each scala, the SV and ST pressure are obtained simultaneously, and the true complex differential pressure can be calculated. Then, a correction for the rise and fall time was made, with an iterative procedure. Data samples in the first 8 s that deviated more than two times the standard deviation from the mean were detected. If so, all data points until the last deviation were removed. An equivalent procedure was performed for the last 8 s. This correction was made to eliminate the combined effect of small deviations between the start of the stimulus and the start of the measurements, attributed to computer lag and the integration time of the lock-in amplifiers. Next, the data were detrended with the Matlab function ‘detrend’ to remove any drift in the signal. Then, the stimulation measurement data was compared to the dark measurement. For both measurements, the mean $\left(\mu \right)$ and standard deviation $\left(\sigma \right)$ were calculated in the complex domain, resulting in an ellipse in the complex plane where the major and minor axis correspond to the standard deviation of the real $\left(Re\right)$ and imaginary $\left(Im\right)$ parts as visualized is Equation 1:


(1) $$\frac{{\mu_{Re}}^{2}}{{\left(1.5.\sigma_{Re}\right)}^{2}}+\frac{{\mu_{Im}}^{2}}{{\left(1.5.\sigma_{Im}\right)}^{2}}=1$$

The stimulation data were considered indistinguishable from random or silent noise if the two ellipses (data and noise) intersected. Thus, these data were excluded from further analysis. Otherwise, the amplitude and phase of the mean were calculated as the complex difference between the stimulation and dark measurement, and the variance of the amplitude and phase was calculated with the formula of linear perturbation theory based on Taylor expansion (Ver Hoef, 2012).

#### 2.4.4 Correlation between the measurement techniques

The Pearson correlation coefficient was employed to assess the predictive capability of different measurements and a linear regression analysis was performed to obtain the best linear fit between the measurements. To optimize the correlation and linear fit, each measurement is normalized to a reference measurement. The reference measurement was determined with the stimulation location set at the standard Baha location and the stimulation intensity set at 70 dB HL. The specimen and the frequency of the reference measurement were adjusted to match the ones of the measurement to normalize. Equation 2 visualizes this normalization method for the differential intracochlear pressure (P_diff_) with subscripts ii, pp., ss, and ff, respectively, the intensity, position, specimen, and frequency of the measurement to normalize. A similar normalization strategy is applied for both the promontory velocity and the ear canal pressure. Additionally, the analysis was repeated by aligning the reference measurement’s stimulation position with that of the measurement being normalized as visualized in Equation 3. This approach isolates variability solely to the stimulation intensity, serving as a baseline.


(2) $$P_{\mathrm{diff}}^{\mathrm{norm}1}=\frac{P_{\mathrm{diff}}\left(\begin{array}{l} \mathrm{Intensity}=ii,\;\mathrm{Position}=\mathrm{pp},\\ \mathrm{Specimen}=ss,\;\mathrm{Frequency}=\mathrm{ff} \end{array}\right)}{P_{\mathrm{diff}}\left(\begin{array}{l} \mathrm{Intensity}=70\;\mathrm{dB}\;HL,\;\mathrm{Position}\\ =\mathrm{Standard}\;\mathrm{Baha},\;\mathrm{Specimen}=ss,\\ \mathrm{Frequency}=\mathrm{ff} \end{array}\right)}$$

(3) $$P_{\mathrm{diff}}^{\mathrm{norm}2}=\frac{P_{\mathrm{diff}}\left(\begin{array}{l} \mathrm{Intensity}=ii,\;\mathrm{Position}=\mathrm{pp},\\ \mathrm{Specimen}=ss,\;\mathrm{Frequency}=\mathrm{ff} \end{array}\right)}{P_{\mathrm{diff}}\left(\begin{array}{l} \mathrm{Intensity}=70\;\mathrm{dB}\;HL,\;\mathrm{Position}=\mathrm{pp},\\ \mathrm{Specimen}=ss,\;\mathrm{Frequency}=\mathrm{ff} \end{array}\right)}$$

#### 2.4.5 The most efficient stimulation position

Determining the most efficient stimulation position involved assessing the position with the highest amplitude for each of the three measurement techniques: ear canal pressure, promontory velocity, and differential pressure. An analysis of variance (ANOVA) test was conducted for each specimen, frequency, and stimulation to evaluate whether any of the four stimulation positions exhibited a significantly different amplitude of the ear canal pressure, promontory velocity, and differential pressure. To address multiple comparisons, a Bonferroni correction was applied (*n* = 180, considering 6 specimens, 10 frequencies, and 3 stimulation intensities).

In cases where the ANOVA test yielded significant results, a *post hoc t*-test was performed between the position with the largest and the second largest amplitude, employing a Bonferroni correction for multiple comparisons (*n* = 180). If this subsequent test also yielded a significant outcome, the position associated with the highest amplitude was designated the most efficient. Data were grouped based on specimen, stimulation frequency, and intensity, resulting in a percentage representation of the most efficient stimulation position for each measurement technique.

## 3 Results

### 3.1 Control measurements

**Figure 2:**
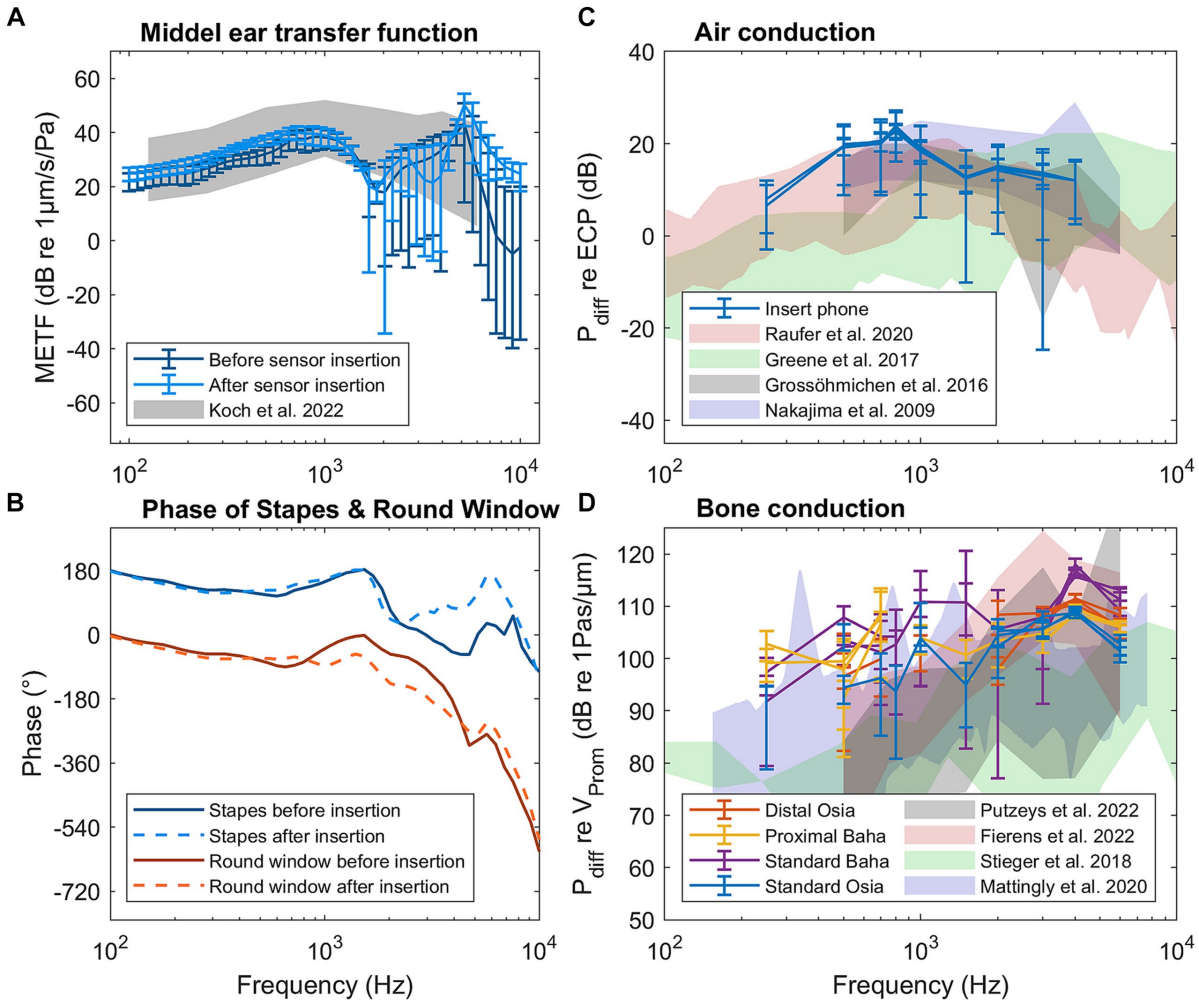
Results of the control measurements for specimen 1L: **(A)** Middle ear transfer function (METF) before and after sensor insertion. **(B)** Phase of the stapes and round window before and after sensor insertion. **(C)** Differential pressure (P_diff_) for air conduction stimulation normalized to ear canal pressure. **(D)** Differential pressure for bone conduction normalized to promontory velocity.

The middle ear transfer functions of one specimen (1L) before and after the insertion of pressure sensors in the cochlea are shown in Figure 2A, while the transfer function of all specimens can be found in Supplementary Figure S1. For 97% of the measurements, the 95% confidence interval overlaps with the range published by Koch et al. (2022), indicating that the specimens are representative of the population. For only one single frequency in one specimen (4L at 1526 Hz), a significant difference was found between the measurements before and after sensor insertion. If the sensor insertion changes the mechanical properties of the hearing organ, differences at multiple frequencies are expected. Therefore, the specimen was not excluded.

Figure 2B shows the phase of the stapes and round window velocity, both before and after sensor insertion for specimen 1L. The phase of all specimens is shown in Supplementary Figure S2. Up to 1 kHz, the phase difference is approximately 180° for all specimens. Above 1 kHz, the phase difference decreases, but the individual phases of the stapes and round window velocity do not change after the sensor insertion.

The differential pressure in the cochlea during pure tone air conduction stimulation normalized to the ear canal pressure is shown in Figure 2C for specimen 1L and in Supplementary Figure S3 for all specimens. At the same time, Figure 2D also shows the differential pressure during bone conduction stimulation normalized to the promontory velocity for specimen 1L, while Supplementary Figure S4 shows the same pressure for all specimens. Both figures also show the ranges of similar measurements published in the literature (Nakajima et al., 2009; Grossöhmichen et al., 2016; Stieger et al., 2018; Mattingly et al., 2020; Fierens et al., 2022; Putzeys et al., 2022).

### 3.2 Correlation between the measurement techniques

The absolute amplitude and phase are shown for each measurement technique across different specimens, stimulation intensities, stimulation positions, and frequencies in Supplementary Figures S5–S39. The correlation and linear regression analyses, using normalization strategy 1, explained in Equation 2, are summarized in Table 2. Figure 3 visually represents the data, depicting the best linear fit. Both analyses were statistically significant (*p* < 0.001), with the Pearson correlation coefficient indicating a slightly stronger association between promontory velocity and differential pressure (rho = 0.69) compared to ear canal pressure and differential pressure (rho = 0.66).

**Table 2:** Characteristics of the correlation and regression analysis between the differential pressure and both the ear canal pressure and the promontory velocity.

	ECP & P_diff_ normalization 1	ECP & P_diff_ normalization 2	V_Prom_ & P_diff_ normalization 1	V_Prom_ & P_diff_ normalization 2
ρ Pearson	0.66	0.96	0.69	0.97
P Pearson correlation	<0.0001	<0.0001	<0.0001	<0.0001
Linear fit	P_diff_ = 0.67*ECP - 0.63 dB	P_diff_ = 1.00*ECP + 0.04 dB	P_diff_ = 0.70* V_Prom_ − 3.18 dB	P_diff_ = 0.99* V_Prom_ + 0.08 dB
RMSE	6.85 dB	2.02 dB	6.60 dB	1.94 dB
R^2^	0.43	0.93	0.47	0.93

While normalization method 1 mitigates the variability arising from differences in frequency and specimen, method 2 additionally addresses the variability associated with the stimulation position. With ECP, ear canal pressure; P_diff_, differential pressure; V_Prom_, promontory velocity.

**Figure 3:**
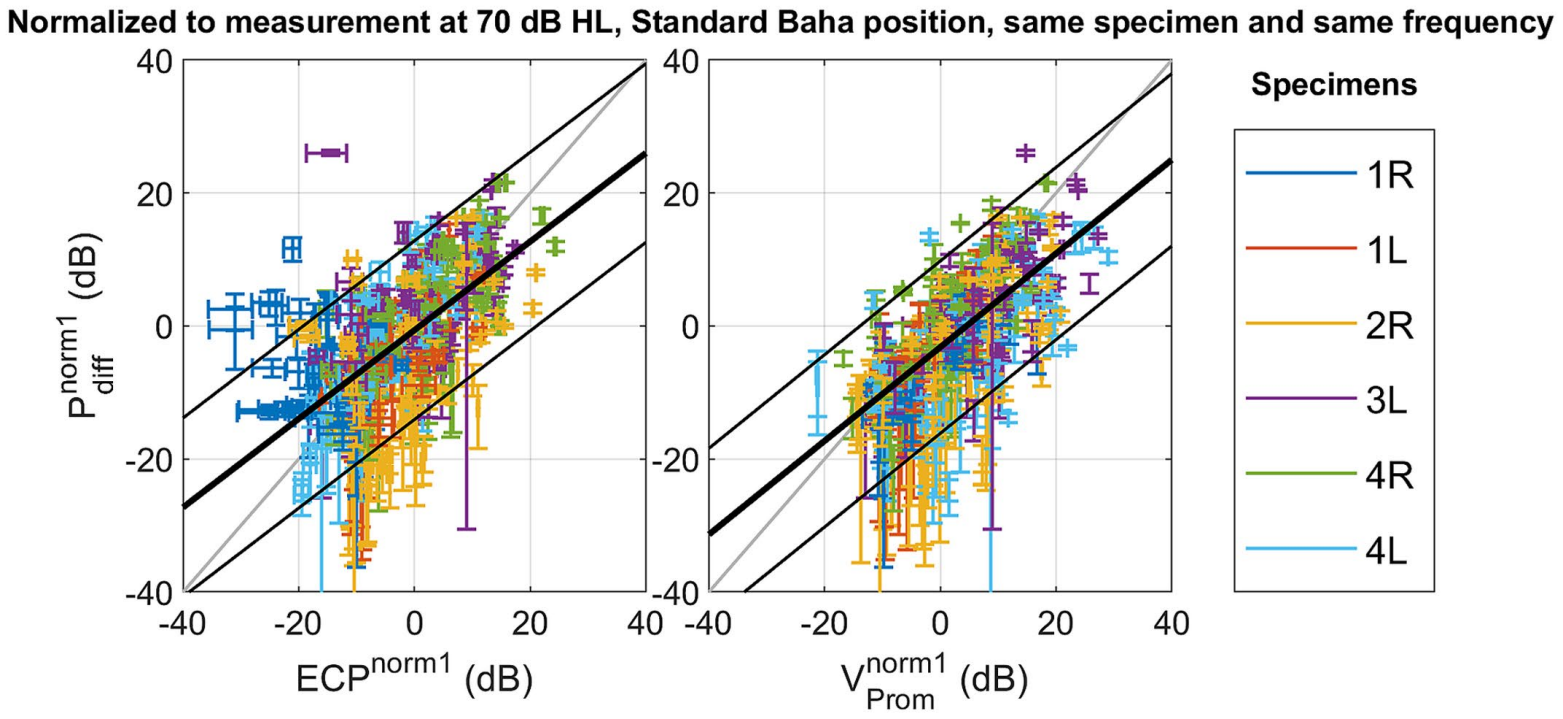
Scatterplot of the differential pressure $\left(P_{\mathrm{diff}}^{\mathrm{norm}1}\right)$ as a function of ear canal pressure $\left(ECP^{\mathrm{norm}1}\right)$ , and as a function of promontory velocity $\left(V_{\mathrm{Prom}}^{\mathrm{norm}1}\right)$ with normalization referencing the measures to a standard condition with the following parameters: stimulation intensity set at 70 dB HL, the stimulation location set as Standard Baha, and the frequency and specimen maintained identical to the measurement under consideration.

**Figure 4:**
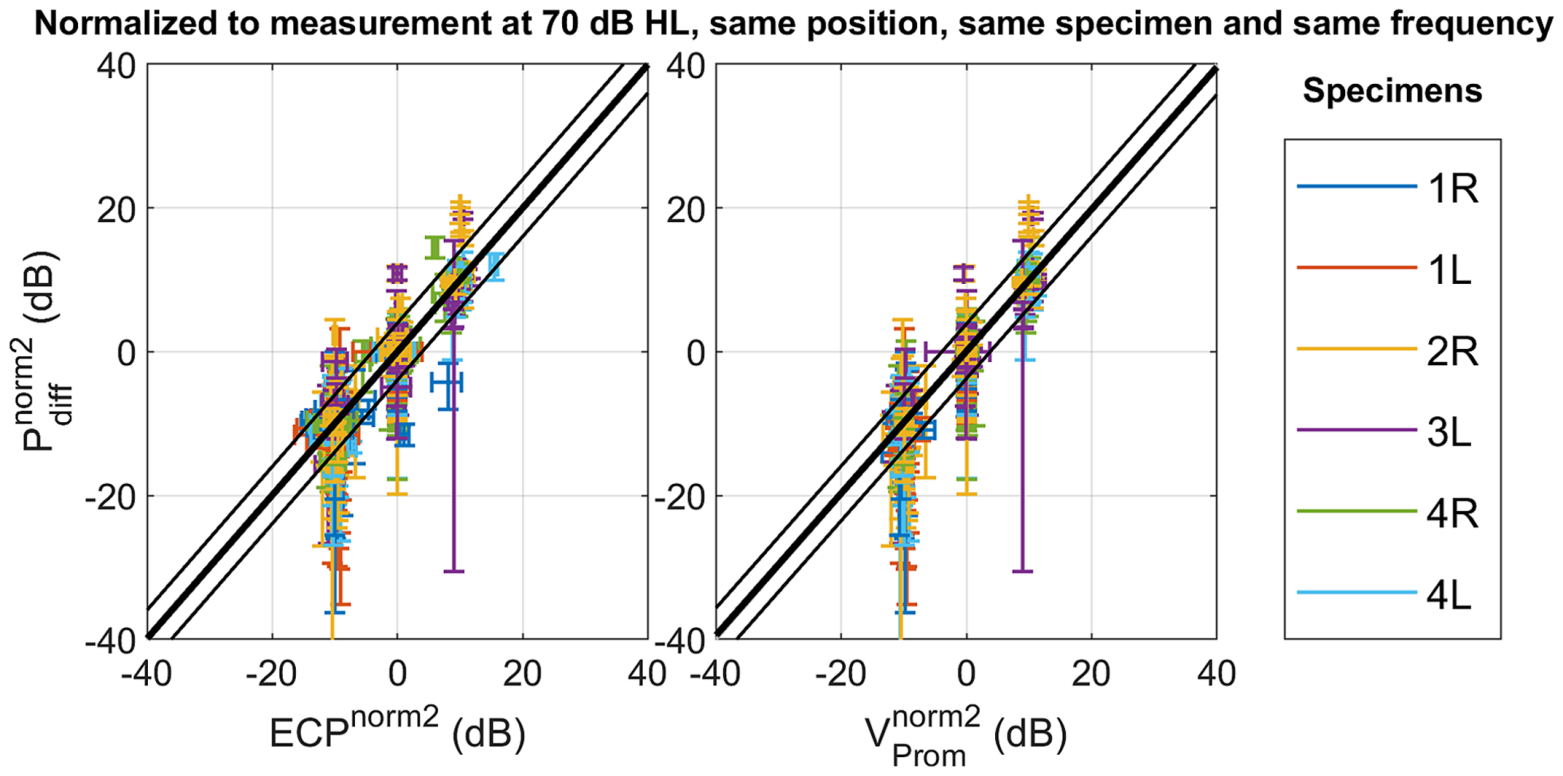
Scatterplot of the differential pressure $\left(P_{\mathrm{diff}}^{\mathrm{norm}2}\right)$ as a function of ear canal pressure $\left(ECP^{\mathrm{norm}2}\right)$ , and as a function of promontory velocity $\left(V_{\mathrm{Prom}}^{\mathrm{norm}2}\right)$ with normalization referencing the measures to a standard condition with the following parameters: stimulation intensity set at 70 dB HL, and the stimulation location, the frequency and specimen maintained identical to the measurement under consideration.

Notably, modifying the normalization method to strategy 2, explained in Equation 3, to mitigate variability arising from alterations in stimulation position, enhances the Pearson correlation coefficient as shown in Figure 4. Similar trends are observed in both the slope of the linear fit and the coefficient of determination. Simultaneously, the root mean square error (RMSE) decreases from 6.85 dB to 2.02 dB for the ear canal pressure and from 6.60 dB to 1.94 dB for the promontory velocity by changing the normalization strategy. The RMSE associated with the second normalization strategy aligns with the test–retest variability observed in pure tone audiometry (Jerlvall et al., 1983) and the one of the test–retest variability of the individual measurements as shown in Supplementary Figure S40. Additionally, the slope of the linear fit with this normalization method approaches unity.

### 3.3 The most efficient stimulation position

Figure 5 illustrates the number of measurements (percentage representation) across specimens, and stimulation frequencies for which each stimulation position generated the highest amplitude for each measurement technique. Depending on the measurement technique, 5–7% of the data was excluded, as it was not significantly above the noise floor. In addition, the results reveal variations in the occurrence of non-significant differences, ranging from 2 to 3%, depending on the measurement. Analysis of the promontory velocity measurements consistently indicates the Standard Osia position as the most efficient – generating the highest amplitude - in most cases. In contrast, Supplementary Figures S13–S27 show a direct comparison of the measurement amplitudes for every specimen, intensity, and frequency, while Supplementary Figures S28–S36 show the equivalent SPL or the level of ear canal pressure that would generate the same intracochlear pressure with an air conduction sound.

**Figure 5:**
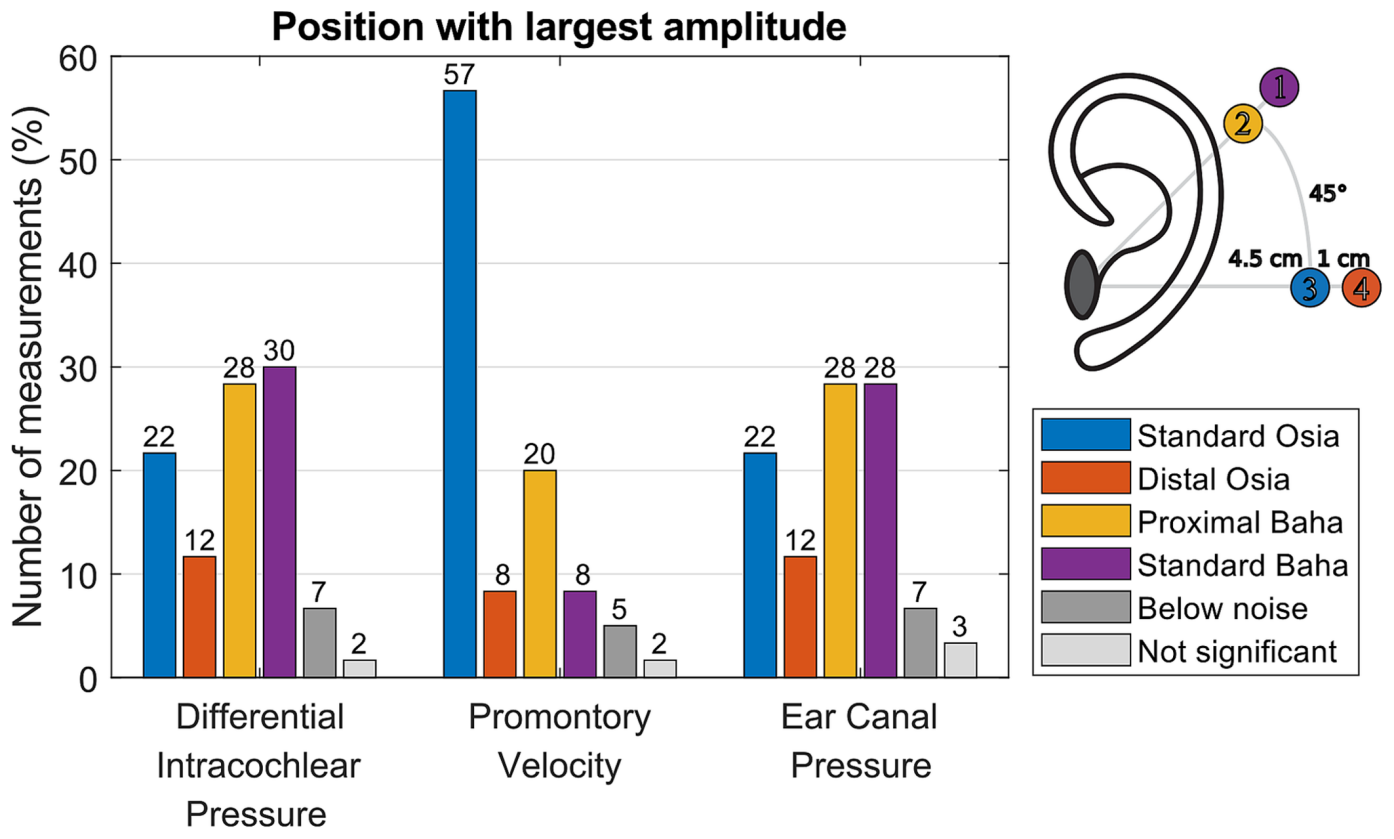
Number of measurements (percentage representation) across specimens, and stimulation frequencies for which each stimulation position generated the highest amplitude for each measurement technique.

The results for ear canal pressure and differential pressure exhibit less consistent patterns. They indicate the Baha positions as the most efficient, with an equal preference for the standard and proximal Baha positions. Supplementary Figure S41 shows the same distribution but for separate frequencies and includes the results for the scala vestibuli and scala tympani pressure. While Figure 5 depicts a comparable distribution for the differential intracochlear and ear canal pressure, Supplementary Figure S41 reveals that considerable variability is observed across frequency.

## 4 Discussion

### 4.1 Correlation between the intracochlear pressure, promontory velocity, and ear canal pressure

With normalization method one – not mitigating the variance of the stimulation position - a linear fit is found between the differential intracochlear pressure and the ear canal pressure, as well as between the differential pressure and the promontory velocity. However, only 43% (for ear canal pressure) and 47% (for promontory velocity) of the variation is explained by the differential pressure, and the RMSE remains 6.60 dB (for promontory velocity) to 6.85 dB (for ear canal pressure). The RMSE surpasses the standard deviation observed in pure tone audiometry (Jerlvall et al., 1983), suggesting that the error in predicting the differential pressure becomes larger than the perceptible difference for an individual.

The differential intracochlear pressure is the measurement technique closest to the organ of Corti. Also, it includes the contributions of all five bone conduction pathways, and previous measurements indicate that it is the most reliable indicator of loudness perception (Borgers et al., 2019; Putzeys et al., 2022; Felix et al., 2023). Therefore, we can conclude that evaluating the loudness perception with the ear canal pressure or promontory velocity introduces a significant 6–7 dB difference, which is larger than the standard deviation in pure tone audiometry (Jerlvall et al., 1983) and the test–retest variability of the individual measures as shown in Supplementary Figure S40. However, as ear canal pressure is a non-invasive measurement technique, it might still be useful in cases where intracochlear pressure measurements are impossible, such as in living patients, and the expected change in stimulation is larger than the introduced error.

Upon removing the stimulation position variation using normalization method two, both the correlation and the slope of the best fit between the ear canal and intracochlear pressure, as well as between promontory velocity and ear canal pressure, tend to approach unity. The percentage of variation explained increases until 93%, and the RMSE decreases until 2.02 dB for ear canal pressure and 1.94 dB for promontory velocity. Ensuring consistent presence throughout all measurements, the foam ear tip from the insert earphone (ER-3C, Etymotic Research, Illinois, USA) remained in the ear canal throughout all measurements and effectively achieved a noise exclusion exceeding 30 dB. Consequently, the likelihood of this correlation being attributed to an acoustically emitted sound from the Baha’s housing transferring to the air conduction pathway is diminished.

The three measurement techniques incorporate varying aspects of the bone conduction pathways, and the fit is enhanced by removing the position-related variation, suggesting that altering the stimulation position also modifies the contributions of the bone conduction pathways. From a clinical perspective, this implies that adjusting the stimulation position can be a valuable strategy to optimize the utilization of intact pathways, depending on the patient’s pathology.

Furthermore, the accurate prediction of intracochlear activity through ear canal pressure enables the assessment of loudness perception in individuals unable to offer feedback, such as children or patients undergoing surgery, with the non-invasive measurement of ear canal pressure. However, the precision of the prediction relies on comparing measurements within the same patient, at the same frequency, and the same stimulation location.

### 4.2 The most efficient stimulation location

The measurement of promontory velocity consistently identifies the standard Osia position as generating the highest amplitude. It is therefore the most efficient stimulation position according to this measure, which aligns with the findings of Fierens et al. (2022). This consistent observation provides further evidence for the precision of this measurement technique (Rosowski et al., 2007; Koch et al., 2022). However, it is essential to note that the velocity is measured in only one dimension as opposed to experiments performed by Dobrev et al. (2016, 2017). As the direction of stimulation at the standard Osia position is most in line with the measurement of the promontory velocity, the one-dimensional measurement may have introduced a bias in the results, potentially leading to an inaccurate estimation of the hearing sensation. Dobrev and Sim (2018) showed that the dominant direction of promontory velocity is specimen and frequency-dependent. Therefore, one-dimensional measurements, which are accessible in most labs, will inherently have a margin of error.

In contrast, both ear canal and intracochlear pressure measurements yield less consistent results regarding the most efficient stimulation position. A plausible interpretation of these findings is that the optimal stimulation position is both patient-and frequency-specific. Moreover, it is crucial to acknowledge that within the auditory system of a living patient, soft sounds are subject to amplification due to the electromobility of outer hair cells, while loud sounds are naturally dampened as a result of the stapedial reflex. This study’s measurements were carried out in cadaveric heads, thus lacking the influence of these physiological phenomena, which could potentially impact the obtained results.

### 4.3 Control measurements

Most of the measurements obtained from the middle ear transfer functions fall within the ranges reported by Koch et al. (2022), indicating that the specimens used in this study are representative of the population. Notably, apart from one specimen at a specific frequency, no significant differences were found between the measurements obtained before and after sensor insertion. This finding provides strong evidence that the insertion of the sensor did not alter the mechanical properties of the ear.

Furthermore, the phase difference observed between stapes and round window velocity, which remains close to 180° up to 1 kHz, confirms the absence of air bubbles in the cochlea and indicates that the fixation method did not reinforce any fluid outlets (Kringlebotn, 1995; Stenfelt et al., 2004).

The measurements of intracochlear pressure normalized to ear canal pressure for air conduction and promontory velocity for bone conduction align with the ranges reported in the literature (Nakajima et al., 2009; Grossöhmichen et al., 2016; Greene et al., 2017; Stieger et al., 2018; Mattingly et al., 2020; Fierens et al., 2022; Putzeys et al., 2022). This alignment validates the intracochlear measurement technique employed in this study.

## 5 Conclusion

The correlation analysis revealed that evaluating bone conduction implants with the ear canal pressure or promontory velocity introduces a 6–7dB difference. This disparity arises from the frequency and sample-specific anatomy dependence of the multiple bone conduction pathways. Assuming intracochlear pressure accurately represents the cochlear drive during bone conduction, approximating via either ear canal pressure or promontory velocity introduces a margin of error.

Furthermore, adjusting the normalization strategy of this analysis showed that altering the stimulation position modifies the contributions of the bone conduction pathways. This underscores the significance of adjusting stimulation position as a valuable strategy to optimize the utilization of intact pathways, contingent upon the patient’s pathology. Additionally, for individuals incapable of providing feedback, the non-invasive measurement of ear canal pressure emerges as a valuable predictor of perceived loudness, as long as comparisons are maintained within the same patient, frequency, and stimulation position.

The measurement of promontory velocity consistently identifies the standard Osia position as the most efficient stimulation position. In contrast, both ear canal and intracochlear pressure measurements yield less consistent results regarding the most efficient stimulation position. This finding suggests that the best stimulation position, based on cadaveric tests, is patient-and frequency-specific.

## Data availability statement

The raw data supporting the conclusions of this article will be made available by the authors, without undue reservation.

## Ethics statement

The studies involving humans were approved by Vesalius Institute - Anatomy and Pathology, University of Leuven - KU Leuven, Belgium. The studies were conducted in accordance with the local legislation and institutional requirements. The participants provided their written informed consent to participate in this study.

## Author contributions

IW: Conceptualization, Data curation, Formal analysis, Investigation, Methodology, Visualization, Writing – original draft, Writing – review & editing. AG: Conceptualization, Investigation, Methodology, Writing – review & editing. TP: Conceptualization, Data curation, Investigation, Methodology, Writing – review & editing. GF: Conceptualization, Data curation, Funding acquisition, Methodology, Writing – review & editing. KD: Conceptualization, Supervision, Writing – review & editing. NV: Conceptualization, Funding acquisition, Resources, Supervision, Writing – review & editing.
