# Supplementary material for: Objective preclinical measures for bone conduction implants
Source: Front Neurosci. 2024 Mar 14;18:1324971. doi: 10.3389/fnins.2024.1324971 (PMC10972895; doi:10.3389/fnins.2024.1324971)
Supplement: Supplementary file 1 [file Data_Sheet_1.PDF]

Supplementary Material

1 Control measurements

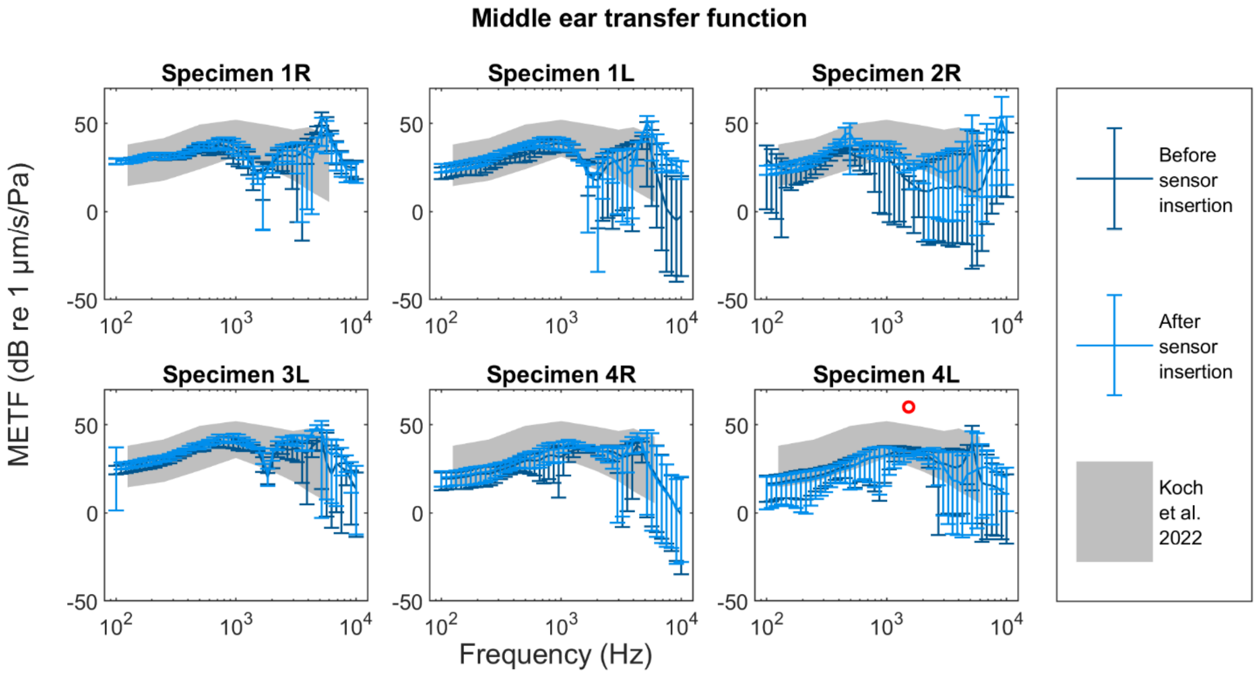

Supplementary Figure 1. Middle ear transfer function before and after sensor insertion for all specimens. For only one single frequency in one specimen (4L at 1526 Hz), a significant difference was found between the measurements before and after sensor insertion. If the sensor insertion changes the mechanical properties of the hearing organ, differences at multiple frequencies are expected. Therefore, the specimen was not excluded.

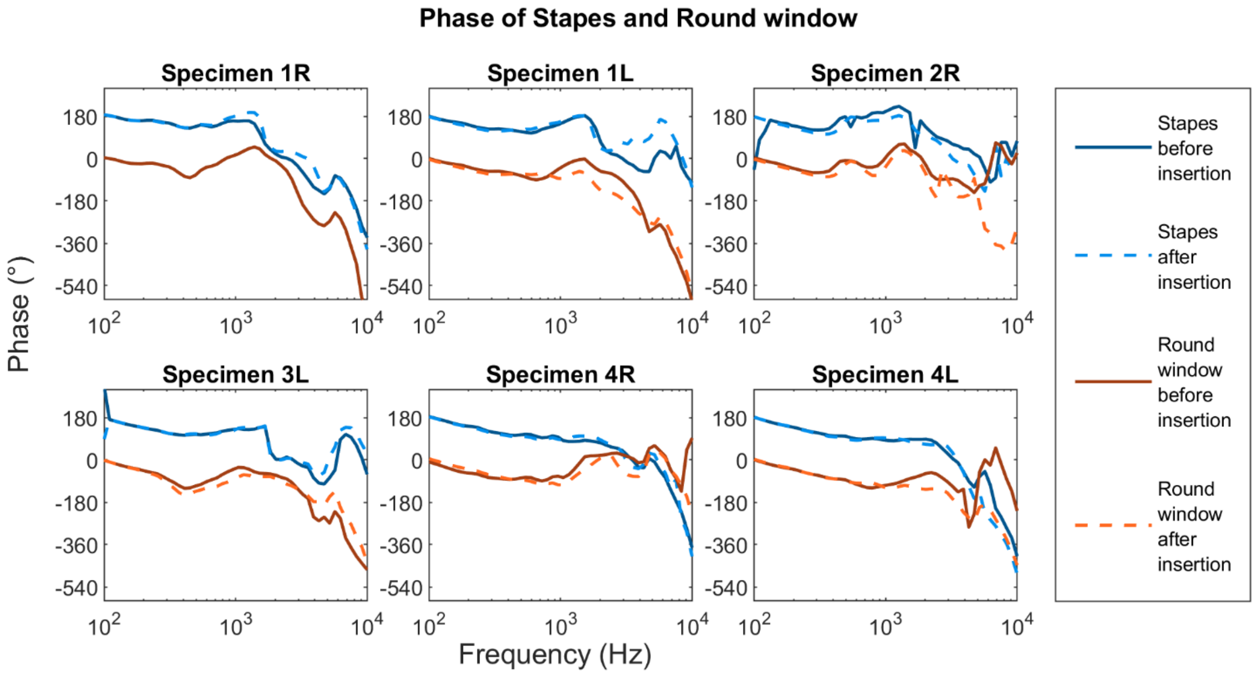

Supplementary Figure 2. Phase of the stapes and round window before and after sensor insertion in all specimens relative to the ear canal pressure. For specimen 1R, a computer problem introduced a time delay between the measurement of the ear canal pressure and the round window velocity. Therefore, this phase data is excluded.

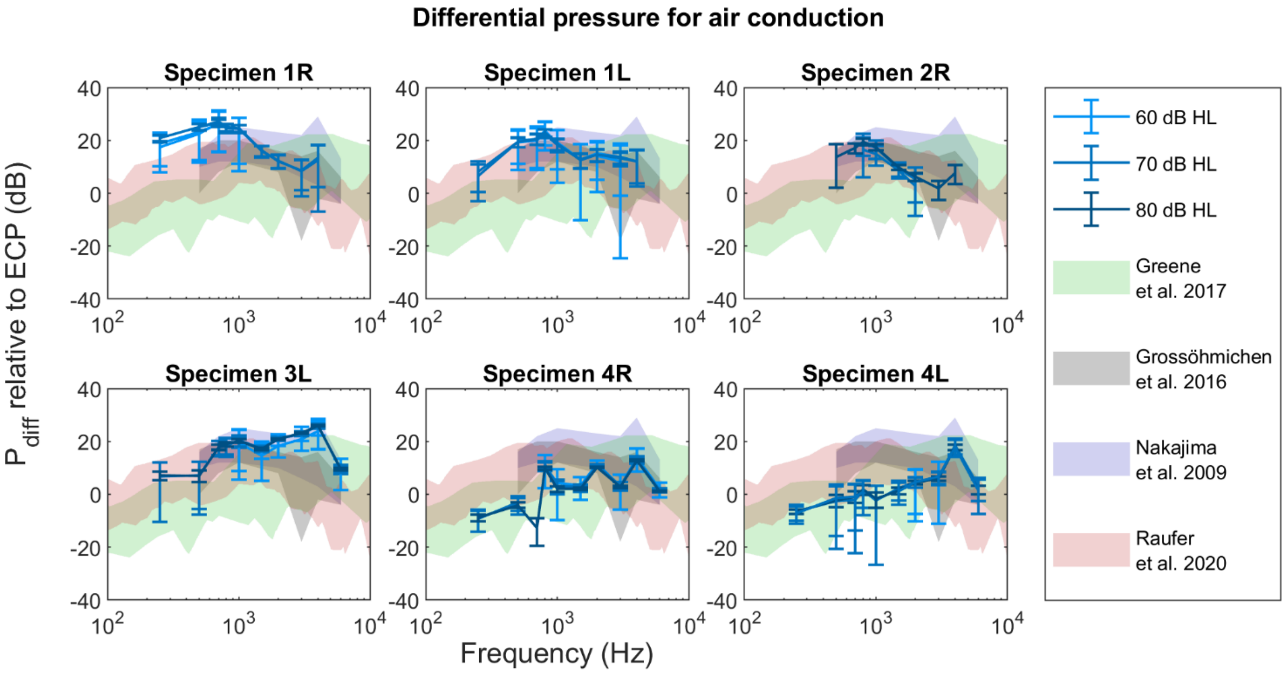

Supplementary Figure 3. Differential pressure for air conduction stimulation normalized to ear canal pressure for all specimens. For specimens 1R and 1L, stimulation at 80 dB HL was not performed.

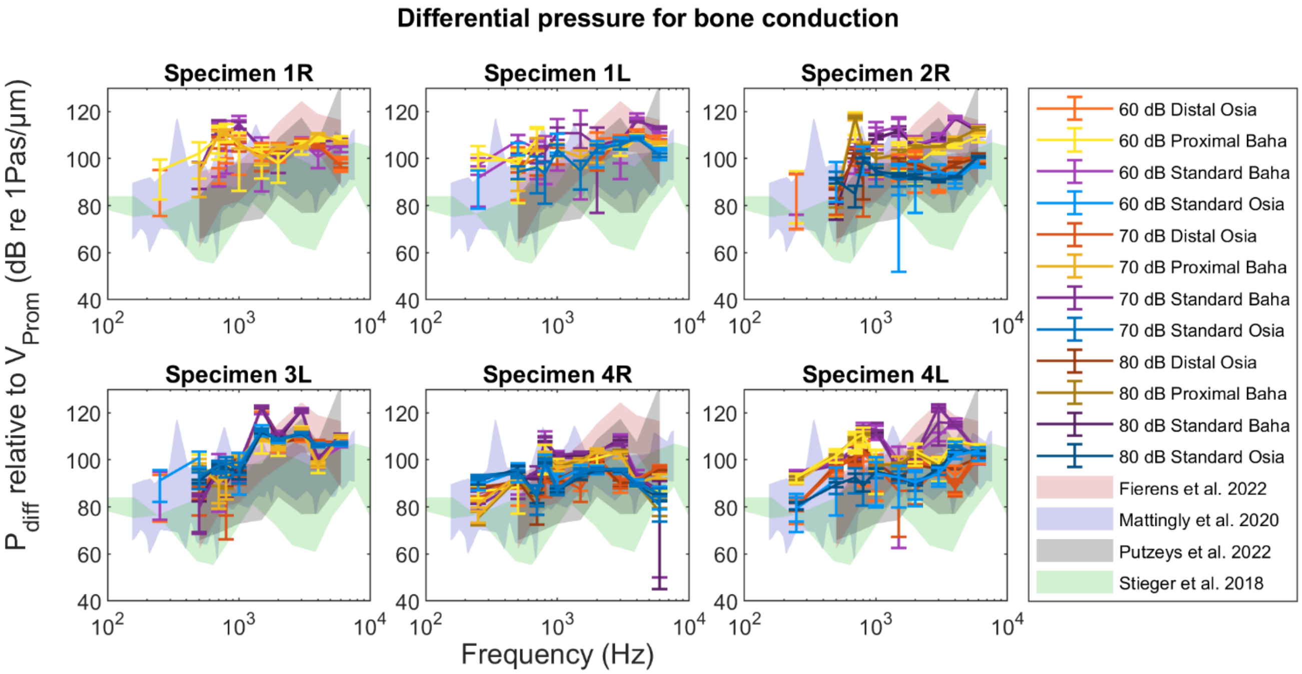

Supplementary Figure 4. Differential pressure for bone conduction stimulation normalized to promontory velocity for all specimens. For specimen 1R, stimulation at 80 dB HL and the Standard Osia position were not performed. For specimen 1L, stimulation at 80 dB HL was not performed.

2 Absolute results for air conduction: all stimulation intensities combined

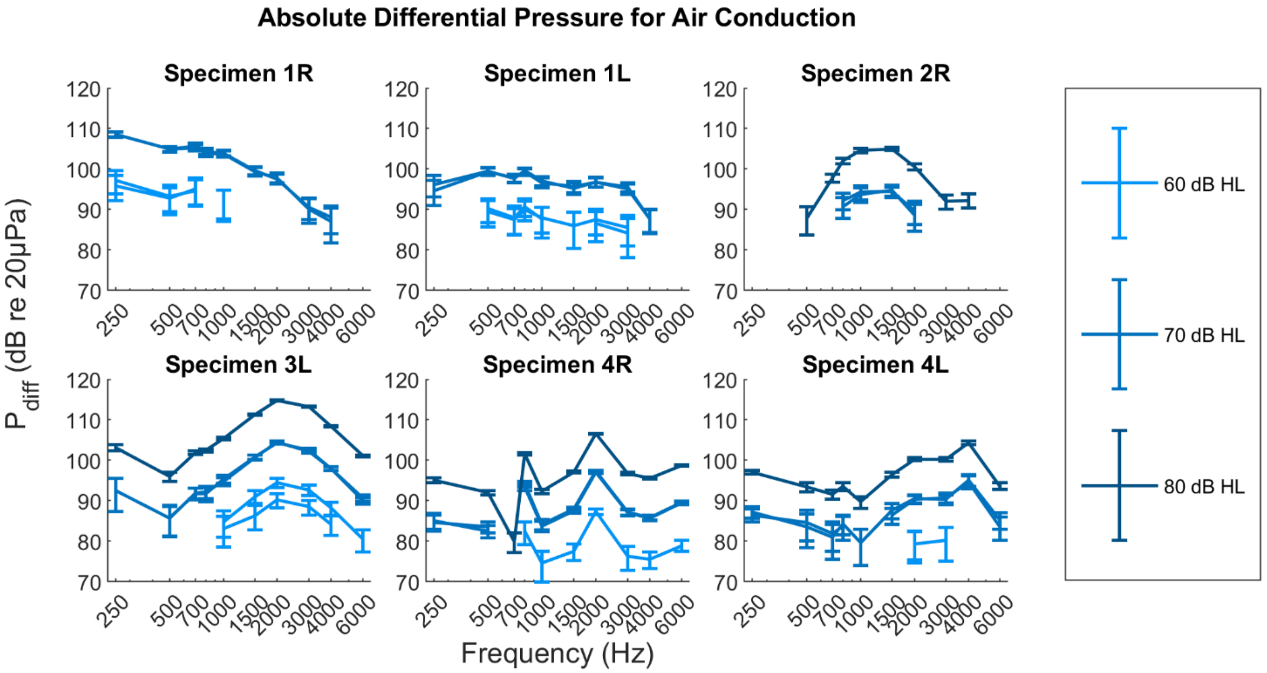

Supplementary Figure 5. Absolute differential pressure for air conduction stimulation for all specimens. For specimens 1R and 1L, stimulation at 80 dB HL was not performed.

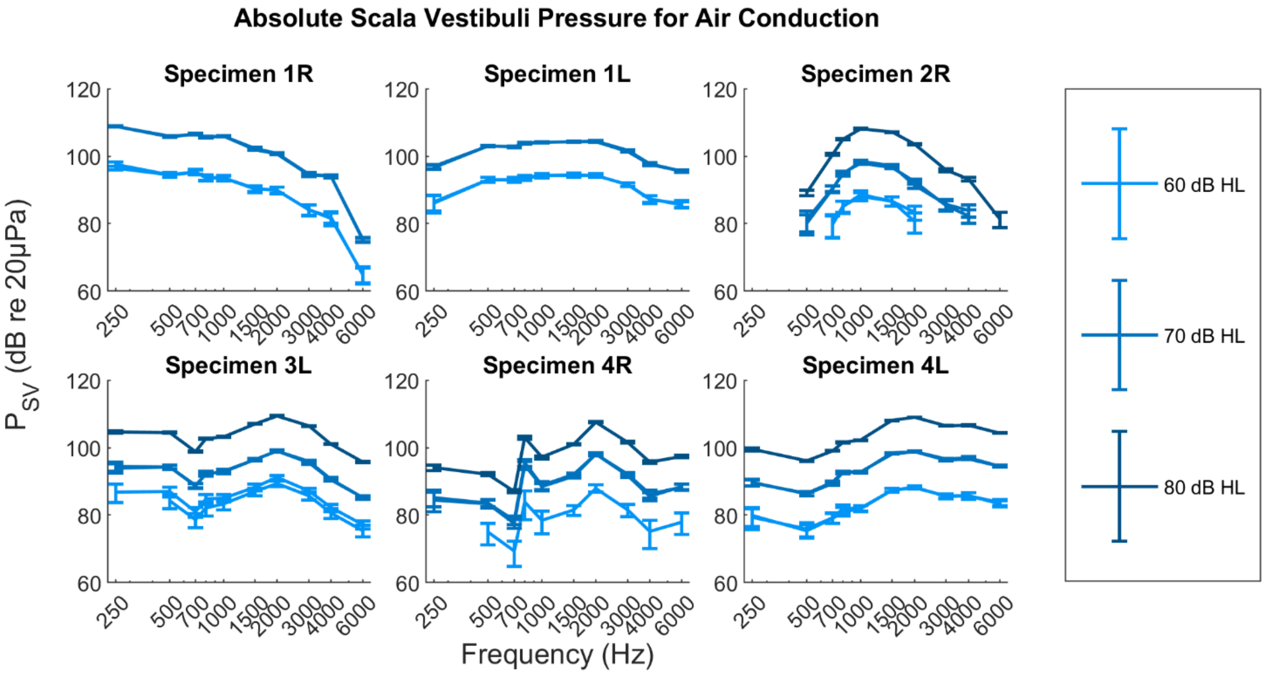

Supplementary Figure 6. Absolute scala vestibuli pressure for air conduction stimulation for all specimens. For specimens 1R and 1L, stimulation at 80 dB HL was not performed.

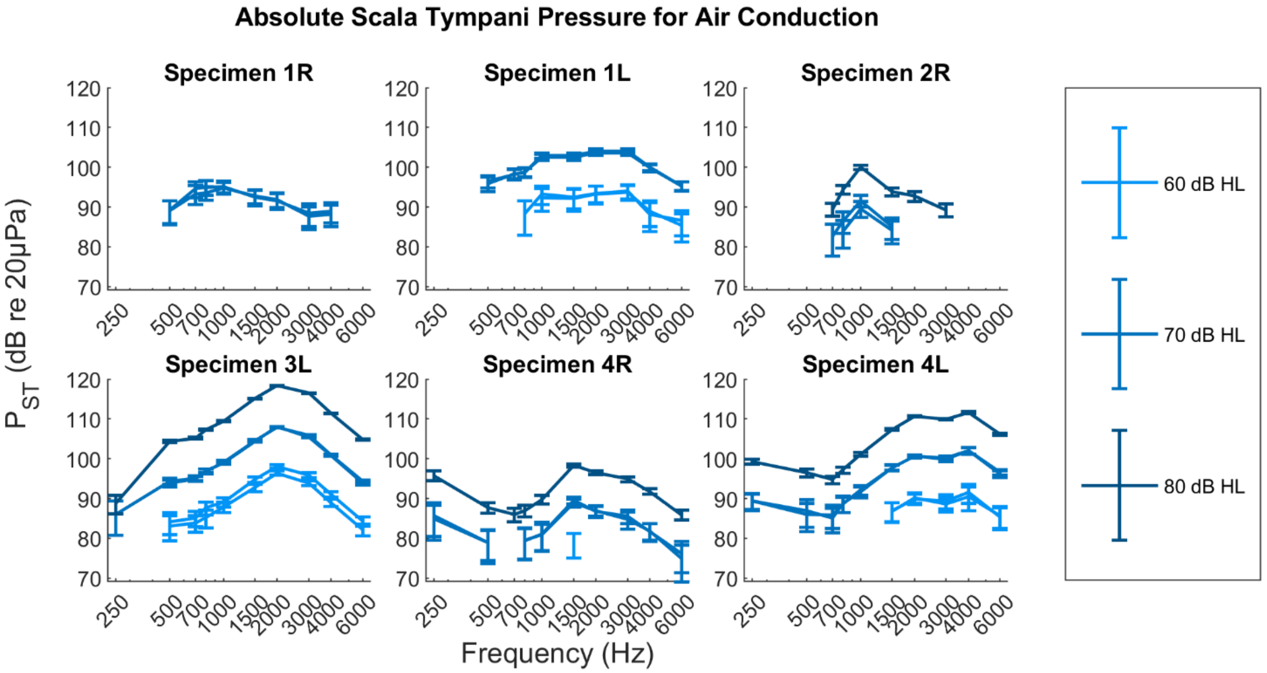

Supplementary Figure 7. Absolute scala tympani pressure for air conduction stimulation for all specimens. For specimens 1R and 1L, stimulation at 80 dB HL was not performed.

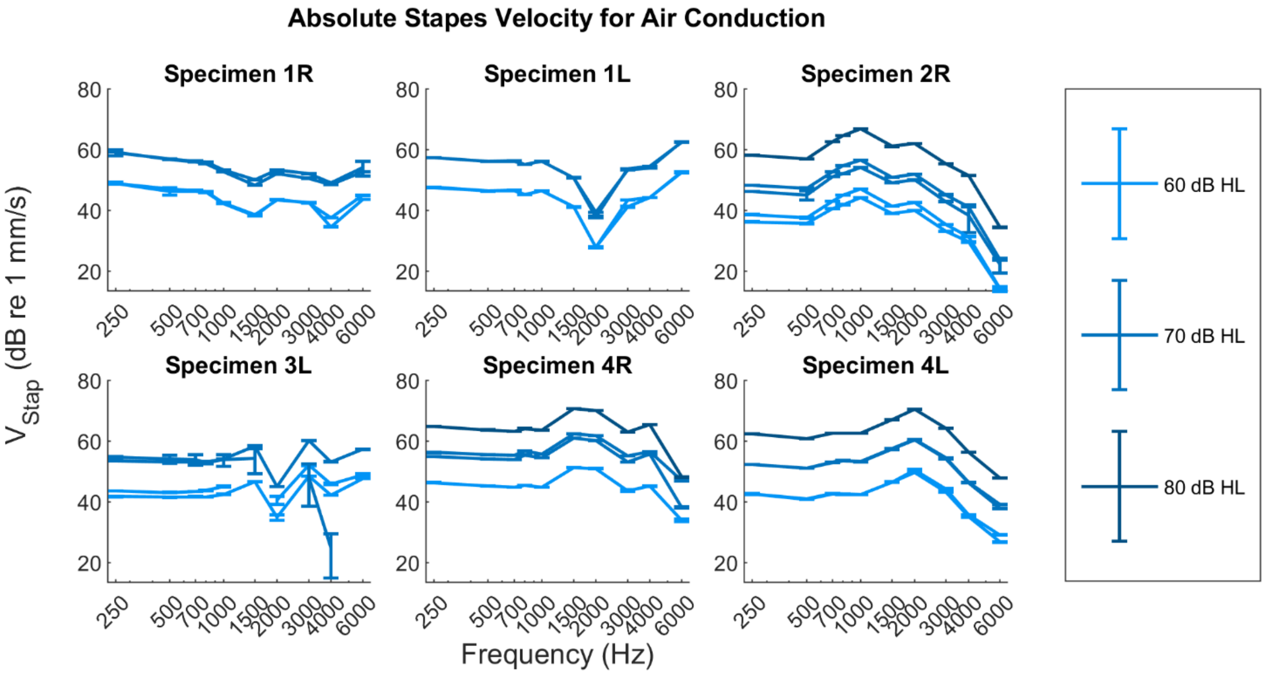

Supplementary Figure 8. Absolute stapes velocity for air conduction stimulation for all specimens. For specimens 1R and 1L, stimulation at 80 dB HL was not performed.

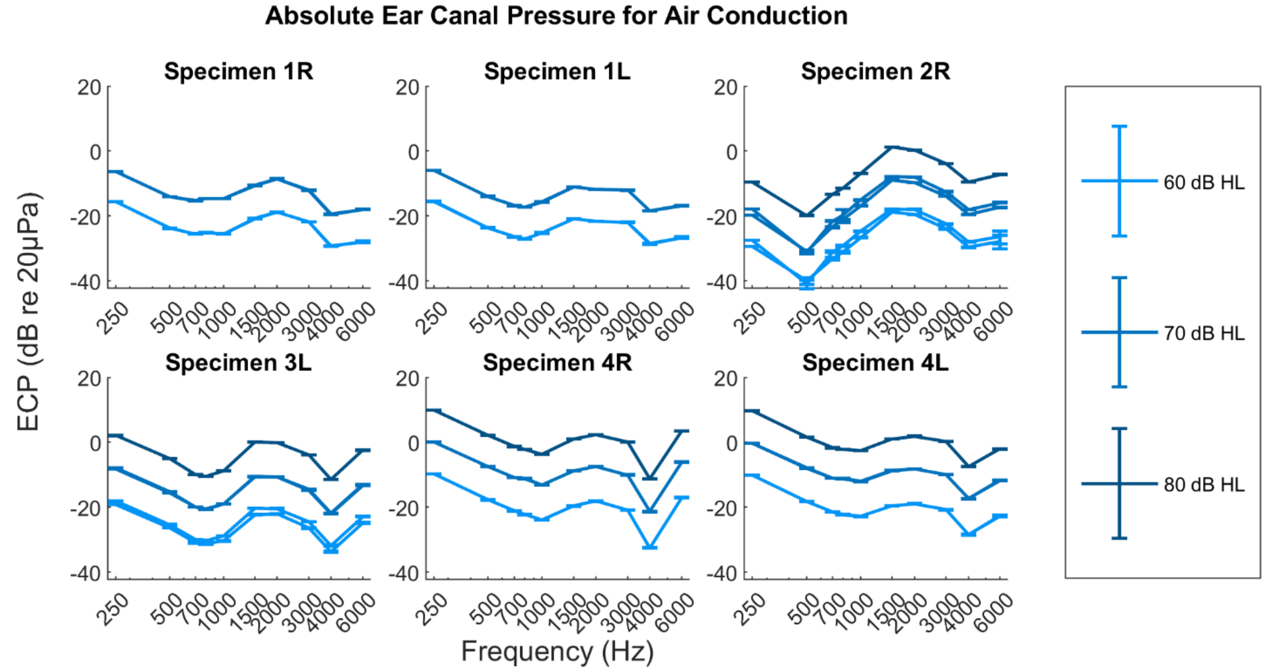

Supplementary Figure 9. Absolute ear canal pressure for air conduction stimulation for all specimens. For specimens 1R and 1L, stimulation at 80 dB HL was not performed.

3 Results for air conduction: phase

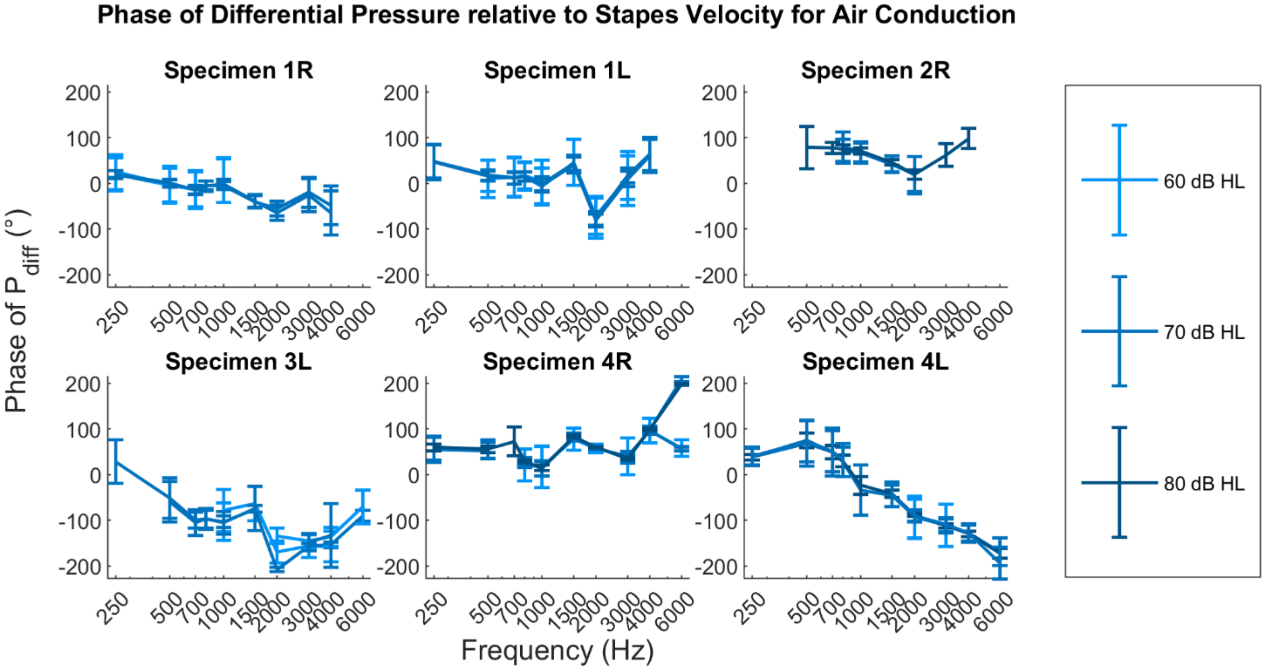

Supplementary Figure 10. The phase of differential pressure for air conduction stimulation in all specimens relative to stapes velocity. For specimens 1R and 1L, stimulation at 80 dB HL was not performed.

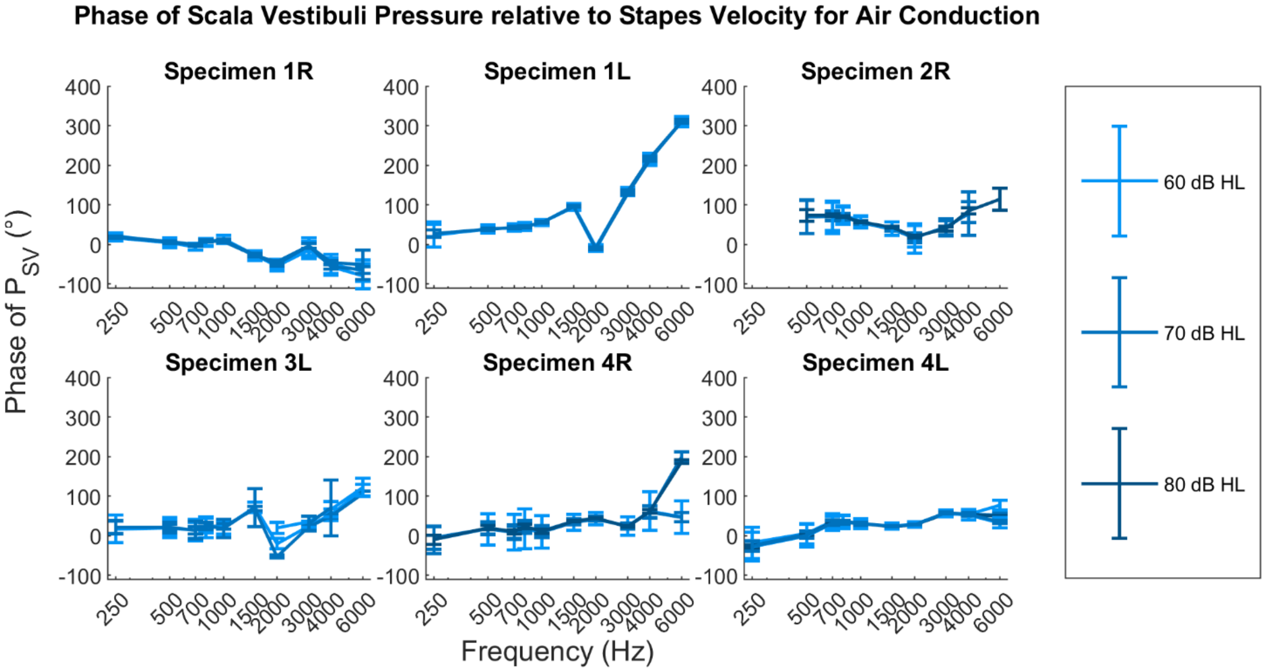

Supplementary Figure 11. The phase of scala vestibuli pressure for air conduction stimulation in all specimens relative to stapes velocity. For specimens 1R and 1L, stimulation at 80 dB HL was not performed.

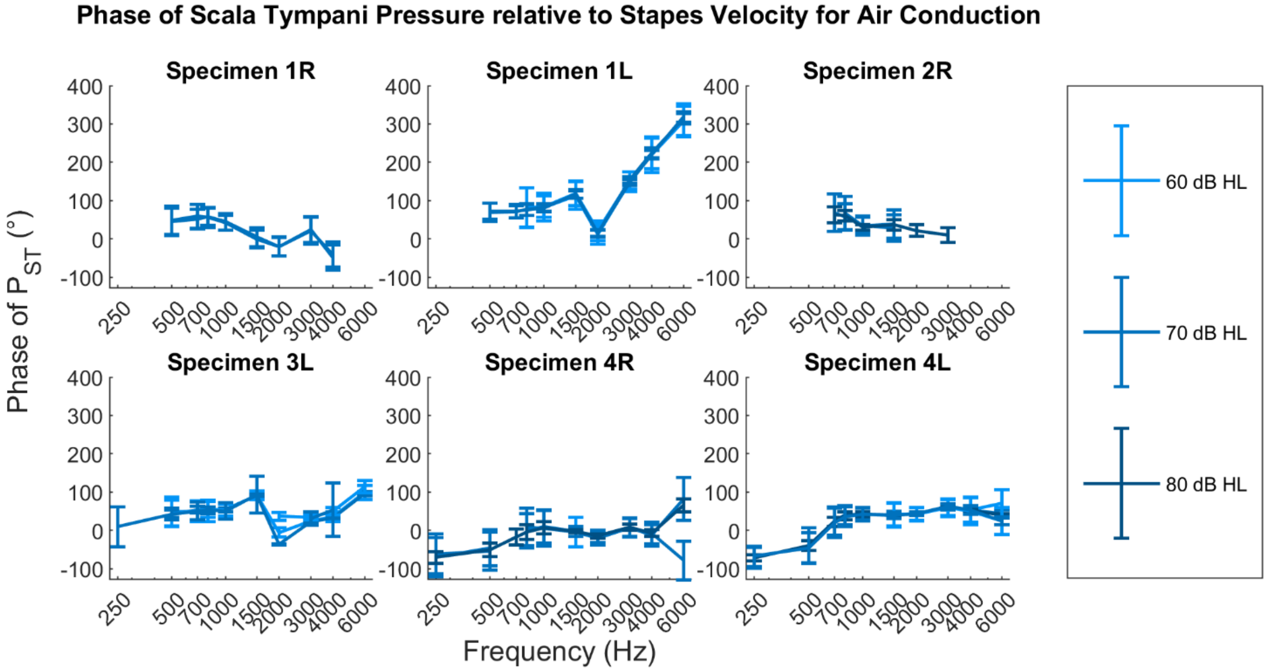

Supplementary Figure 12. The phase of scala tympani pressure for air conduction stimulation in all specimens relative to stapes velocity. For specimens 1R and 1L, stimulation at 80 dB HL was not performed.

4 Absolute results for bone conduction: all stimulation intensities separate

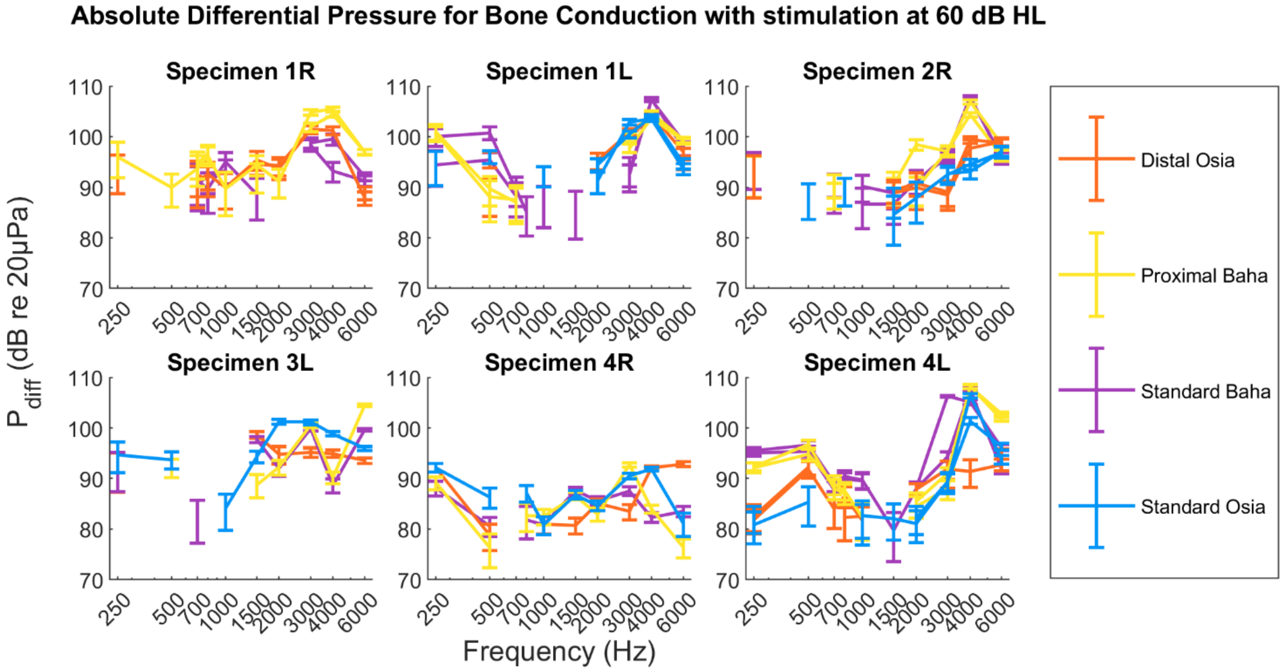

Supplementary Figure 13. Absolute differential pressure for bone conduction stimulation in all specimens with stimulation at 60 dB HL. For specimen 1R, stimulation at the Standard Osia position was not performed.

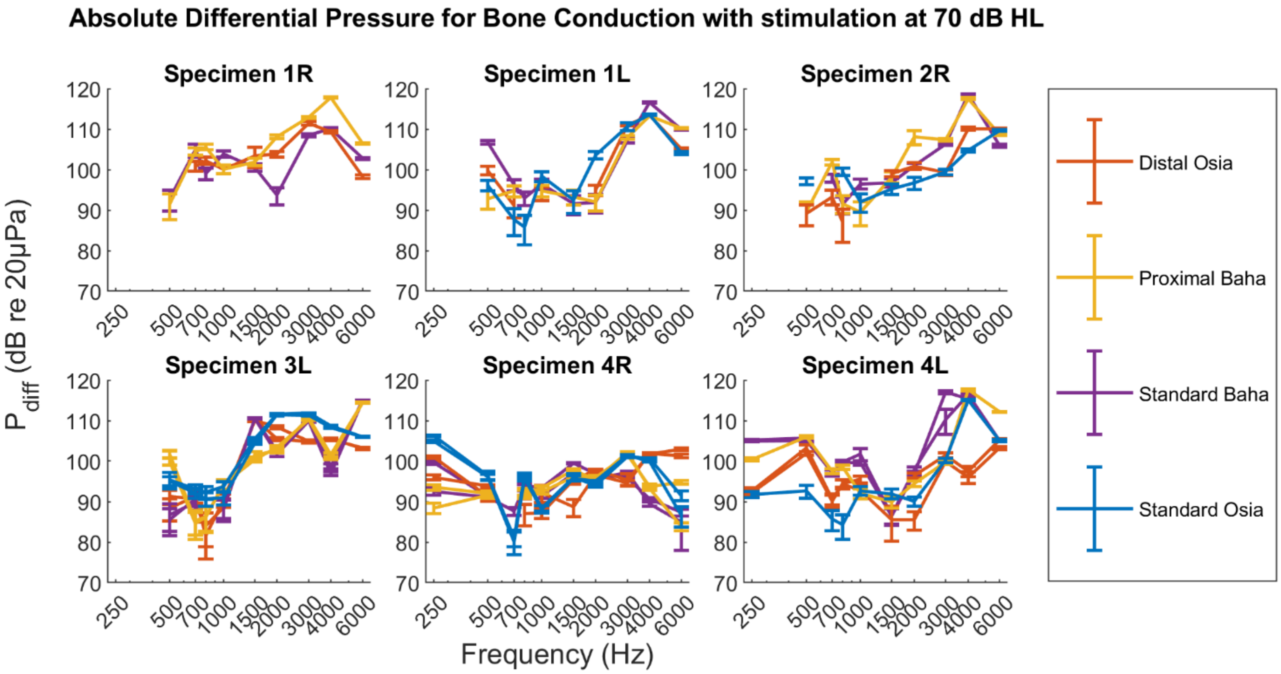

Supplementary Figure 14. Absolute differential pressure for bone conduction stimulation in all specimens with stimulation at 70 dB HL. For specimen 1R, stimulation at the Standard Osia position was not performed.

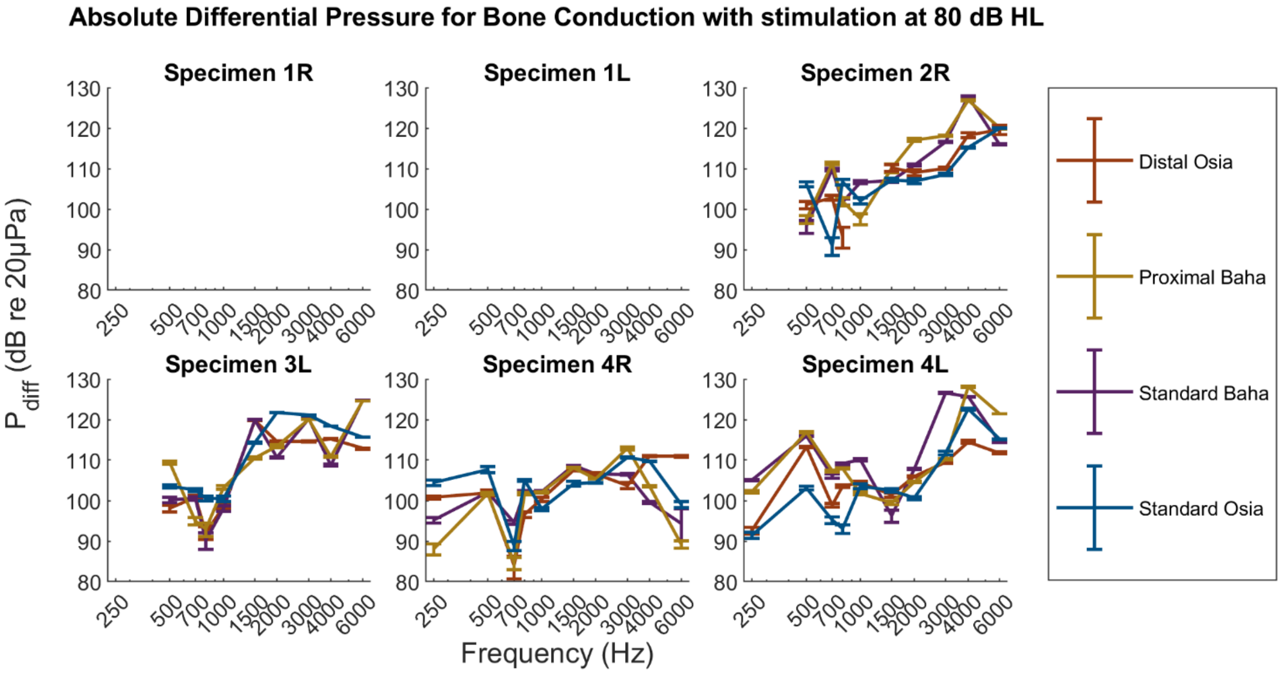

Supplementary Figure 15. Absolute differential pressure for bone conduction stimulation in all specimens with stimulation at 80 dB HL. For specimens 1R and 1L, stimulation at 80 dB HL was not performed.

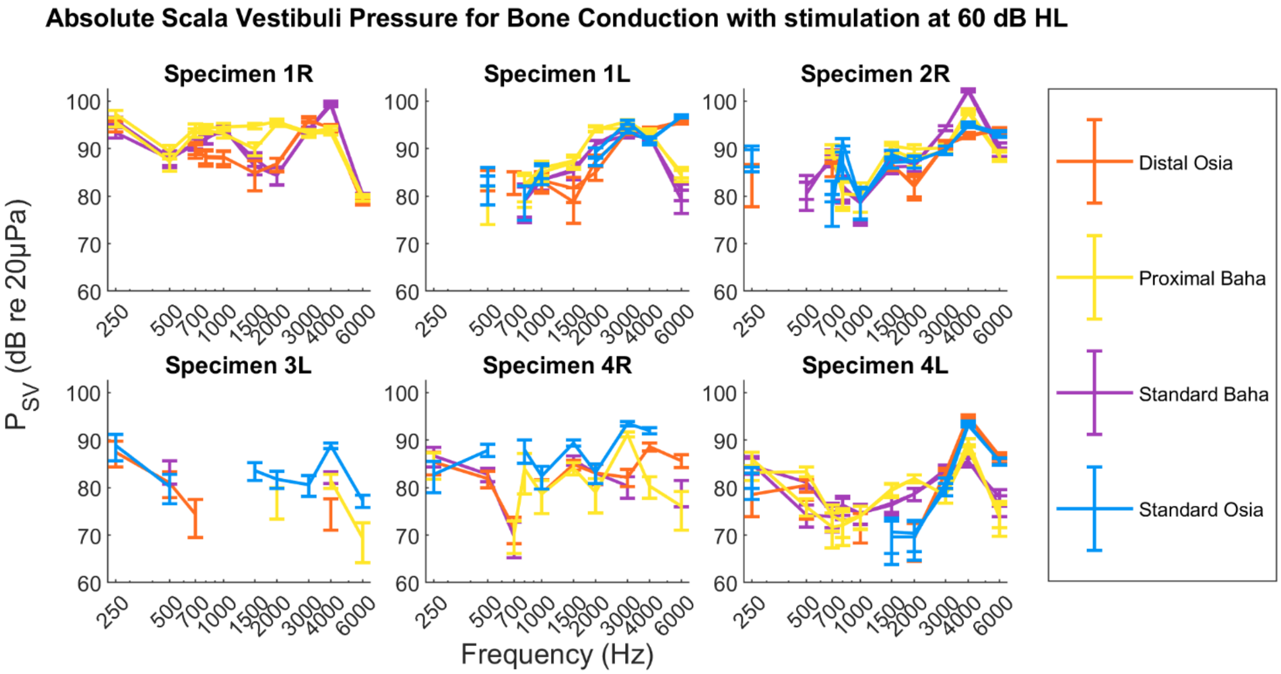

Supplementary Figure 16. Absolute scala vestibuli pressure for bone conduction stimulation in all specimens with stimulation at 60 dB HL. For specimen 1R, stimulation at the Standard Osia position was not performed.

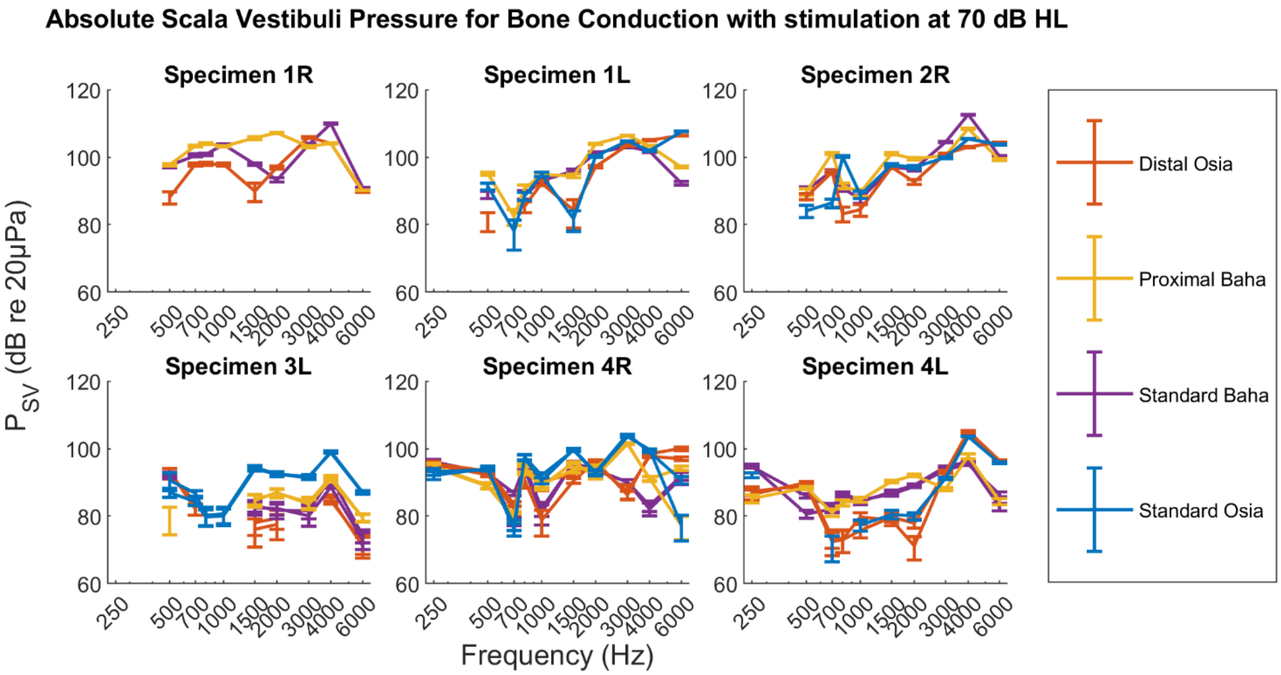

Supplementary Figure 17. Absolute scala vestibuli pressure for bone conduction stimulation in all specimens with stimulation at 70 dB HL. For specimen 1R, stimulation at the Standard Osia position was not performed.

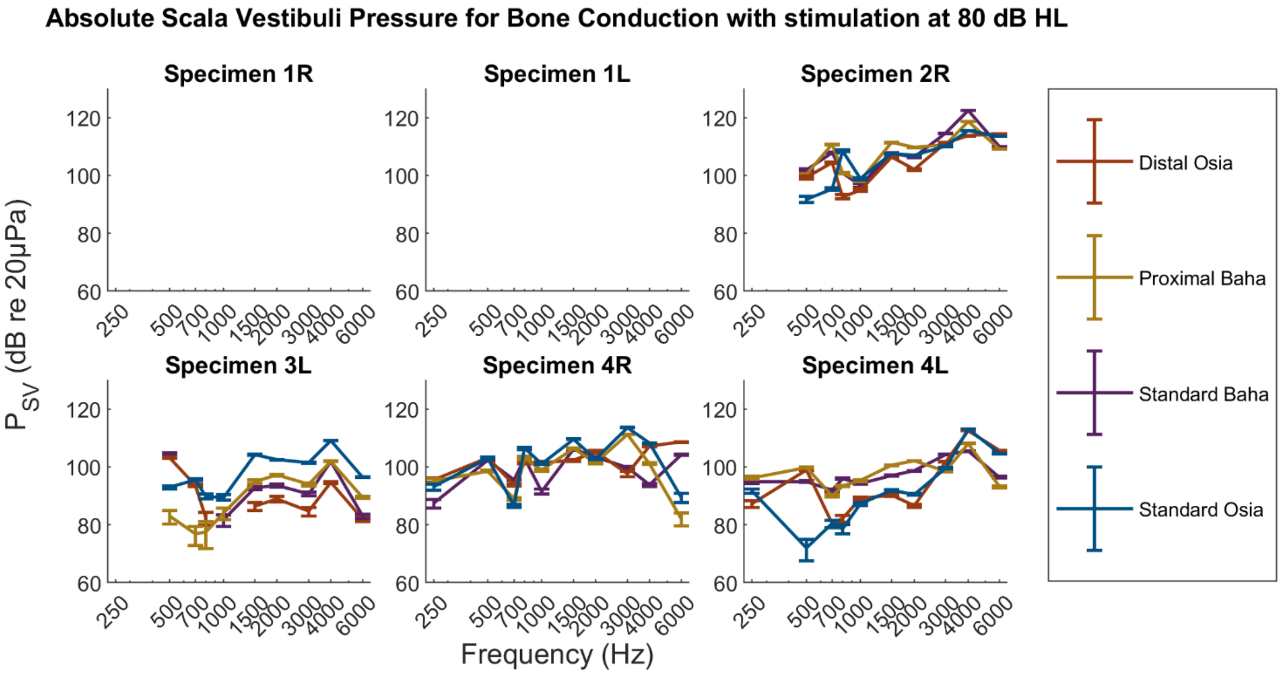

Supplementary Figure 18. Absolute scala vestibuli pressure for bone conduction stimulation in all specimens with stimulation at 80 dB HL. For specimens 1R and 1L, stimulation at 80 dB HL was not performed.

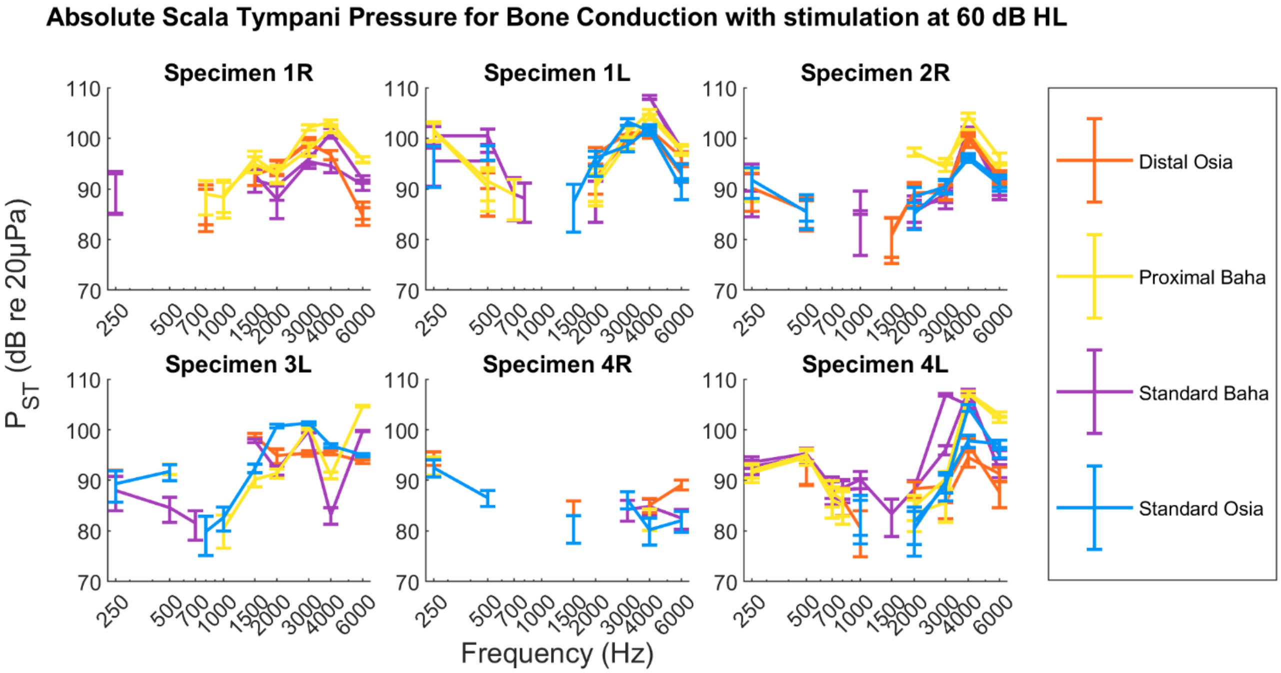

Supplementary Figure 19. Absolute scala tympani pressure for bone conduction stimulation in all specimens with stimulation at 60 dB HL. For specimen 1R, stimulation at the Standard Osia position was not performed.

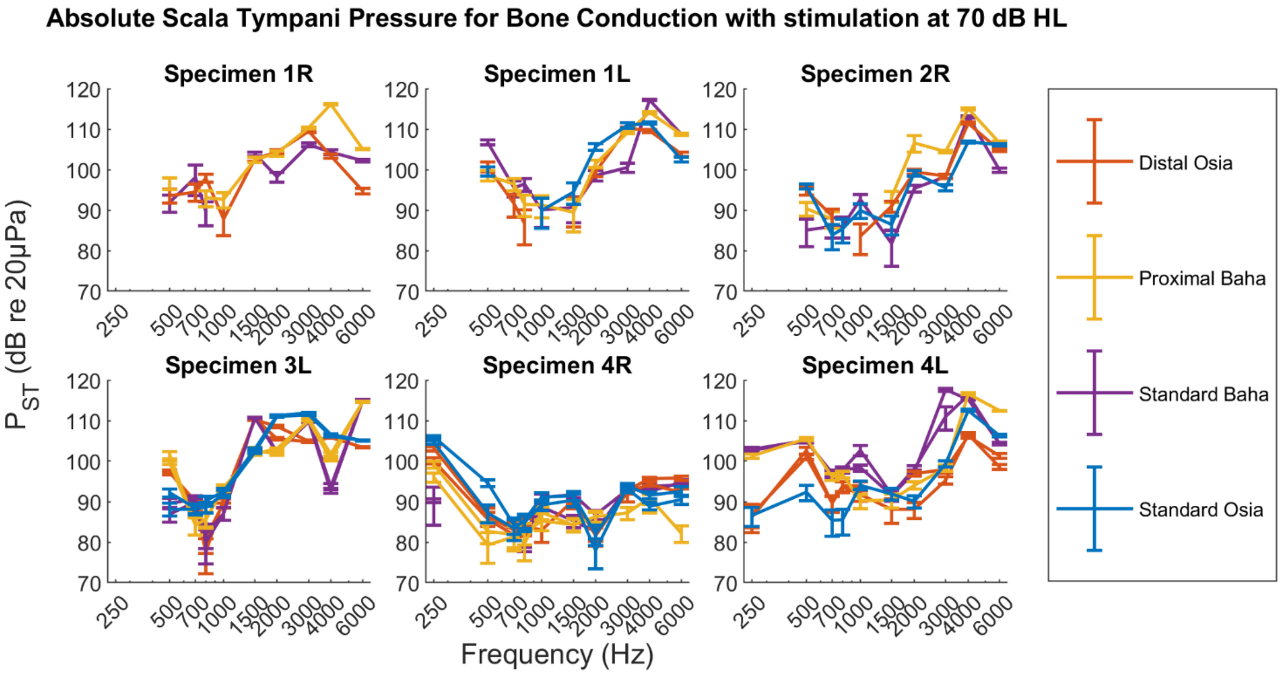

Supplementary Figure 20. Absolute scala tympani pressure for bone conduction stimulation in all specimens with stimulation at 70 dB HL. For specimen 1R, stimulation at the Standard Osia position was not performed.

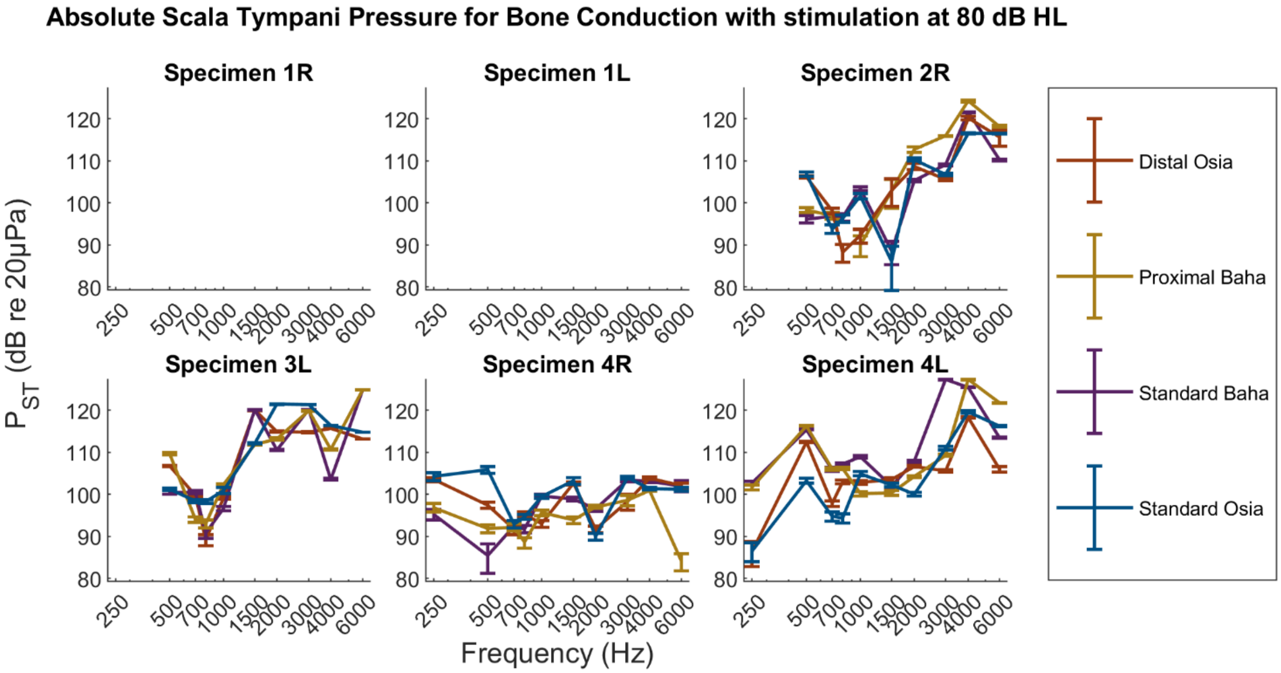

Supplementary Figure 21. Absolute scala tympani pressure for bone conduction stimulation in all specimens with stimulation at 80 dB HL. For specimens 1R and 1L, stimulation at 80 dB HL was not performed.

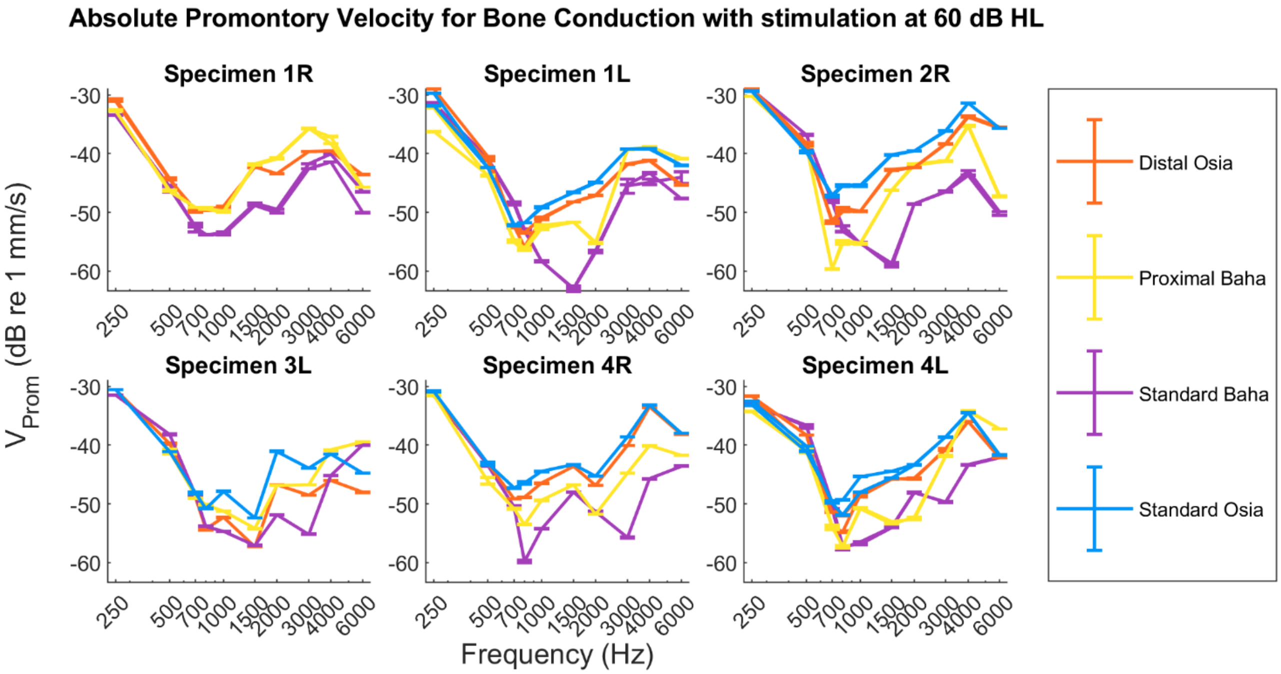

Supplementary Figure 22. Absolute promontory velocity for bone conduction stimulation in all specimens with stimulation at 60 dB HL. For specimen 1R, stimulation at the Standard Osia position was not performed.

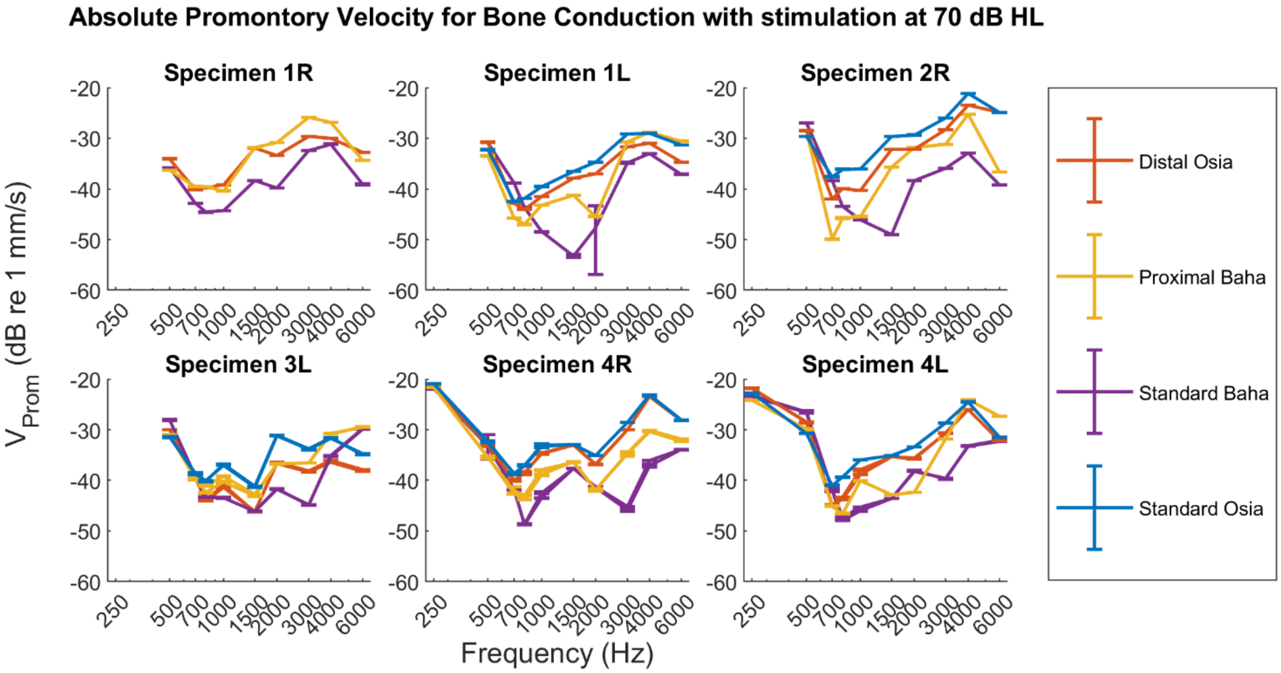

Supplementary Figure 23. Absolute promontory velocity for bone conduction stimulation in all specimens with stimulation at 70 dB HL. For specimen 1R, stimulation at the Standard Osia position was not performed.

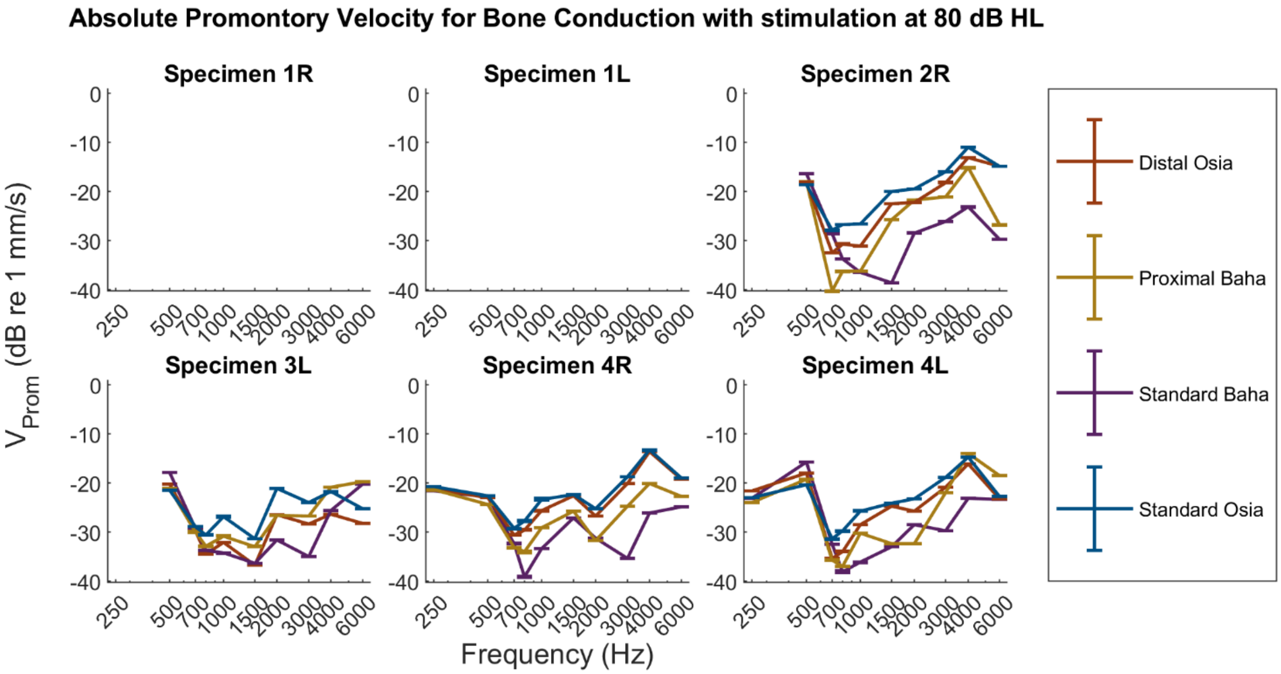

Supplementary Figure 24. Absolute promontory velocity for bone conduction stimulation in all specimens with stimulation at 80 dB HL. For specimens 1R and 1L, stimulation at 80 dB HL was not performed.

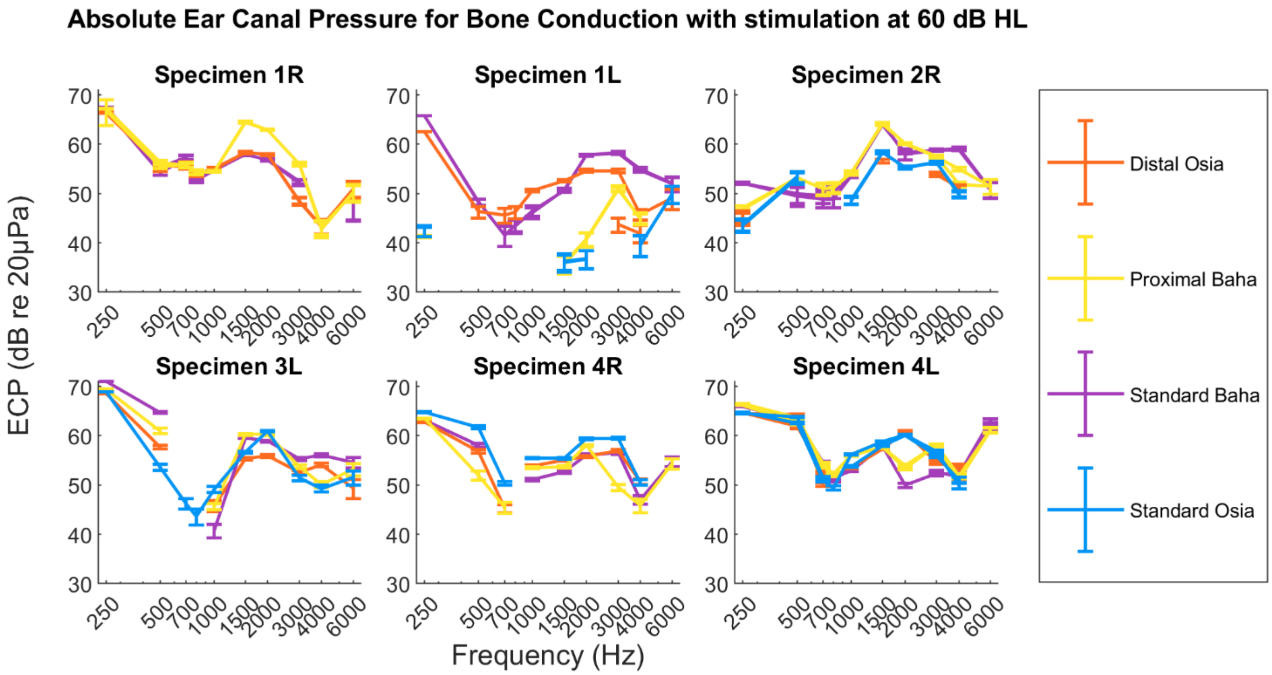

Supplementary Figure 25. Absolute ear canal pressure for bone conduction stimulation in all specimens with stimulation at 60 dB HL. For specimen 1R, stimulation at the Standard Osia position was not performed.

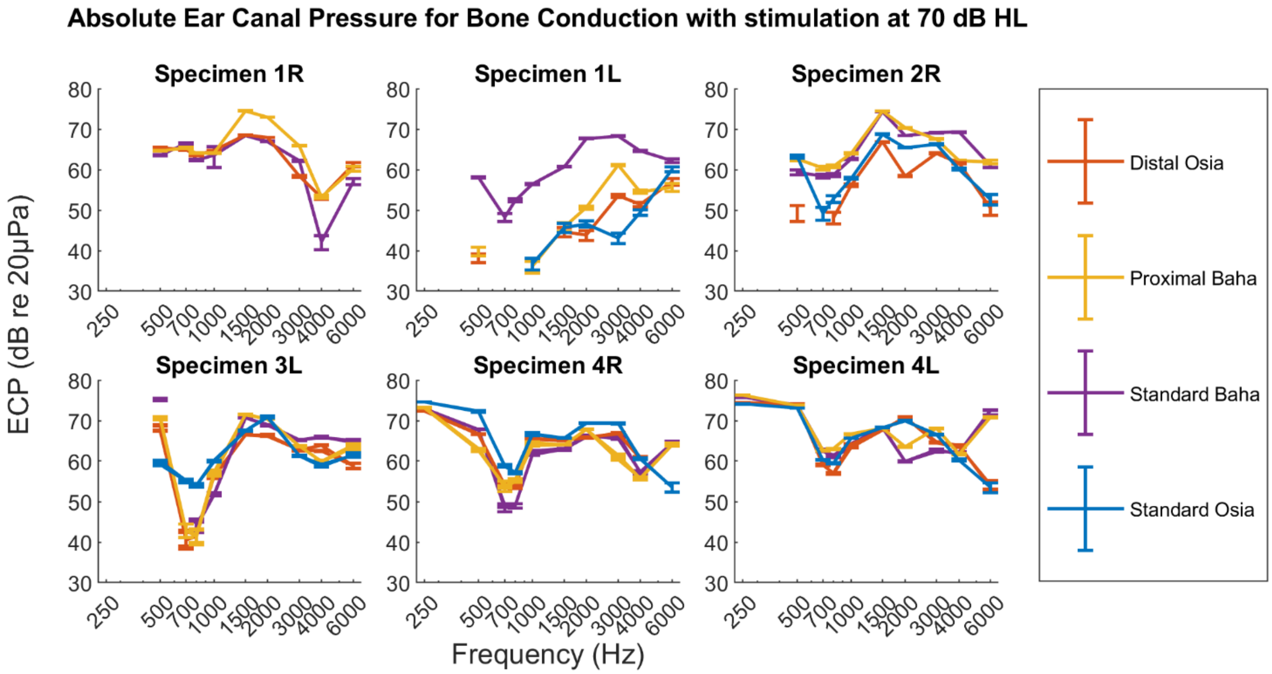

Supplementary Figure 26. Absolute ear canal pressure for bone conduction stimulation in all specimens with stimulation at 70 dB HL. For specimen 1R, stimulation at the Standard Osia position was not performed.

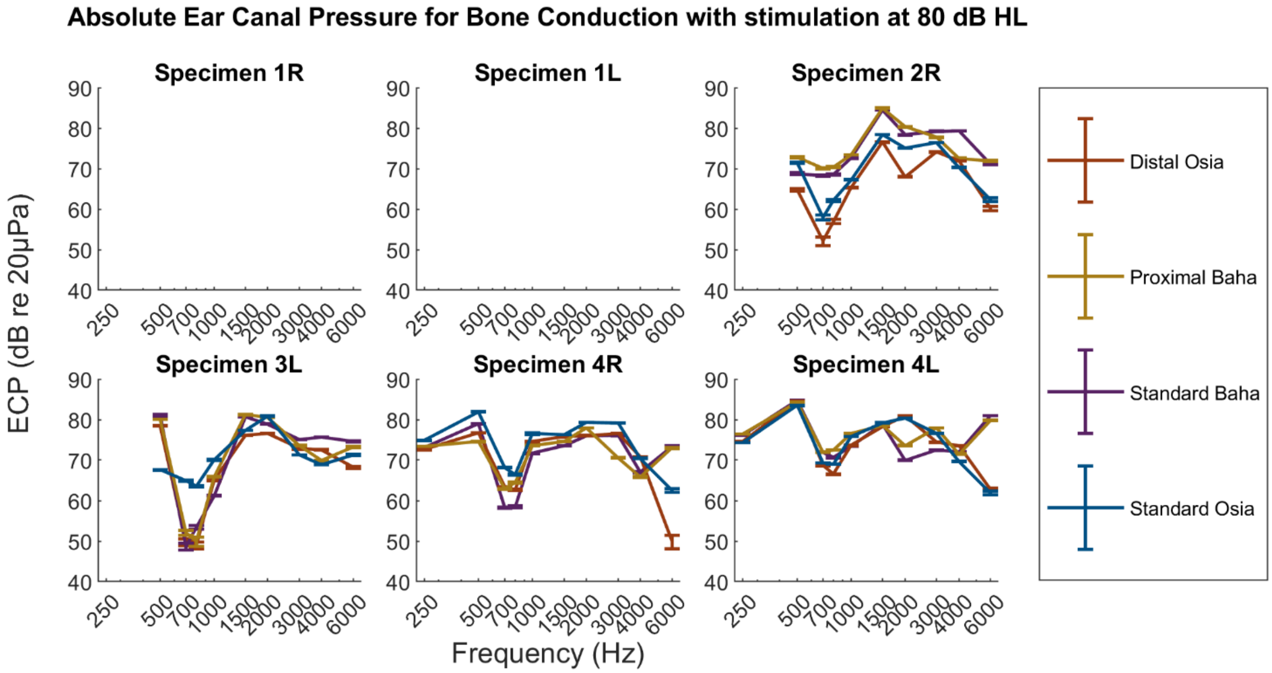

Supplementary Figure 27. Absolute ear canal pressure for bone conduction stimulation in all specimens with stimulation at 80 dB HL. For specimens 1R and 1L, stimulation at 80 dB HL was not performed.

5 Equivalent SPL in the ear canal for bone conduction: all stimulation intensities separate

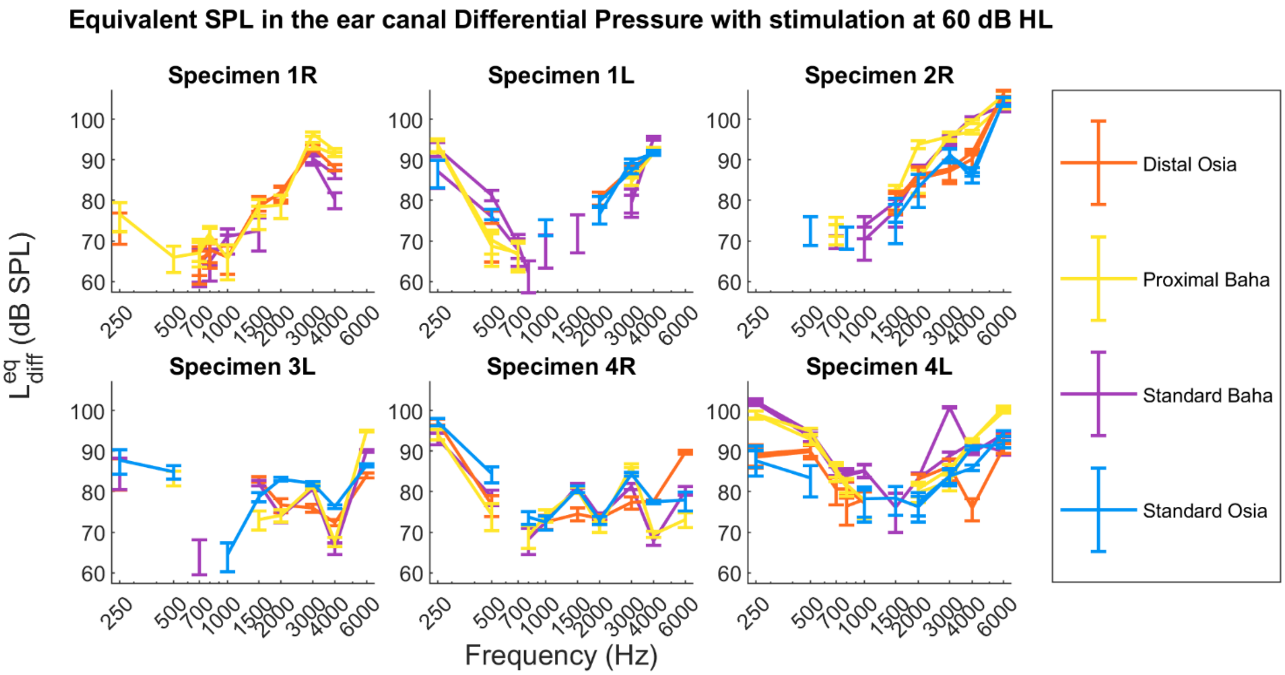

Supplementary Figure 28. Equivalent SPL in the ear canal for differential pressure for bone conduction stimulation in all specimens with stimulation at 60 dB HL. For specimen 1R, stimulation at the Standard Osia position was not performed.

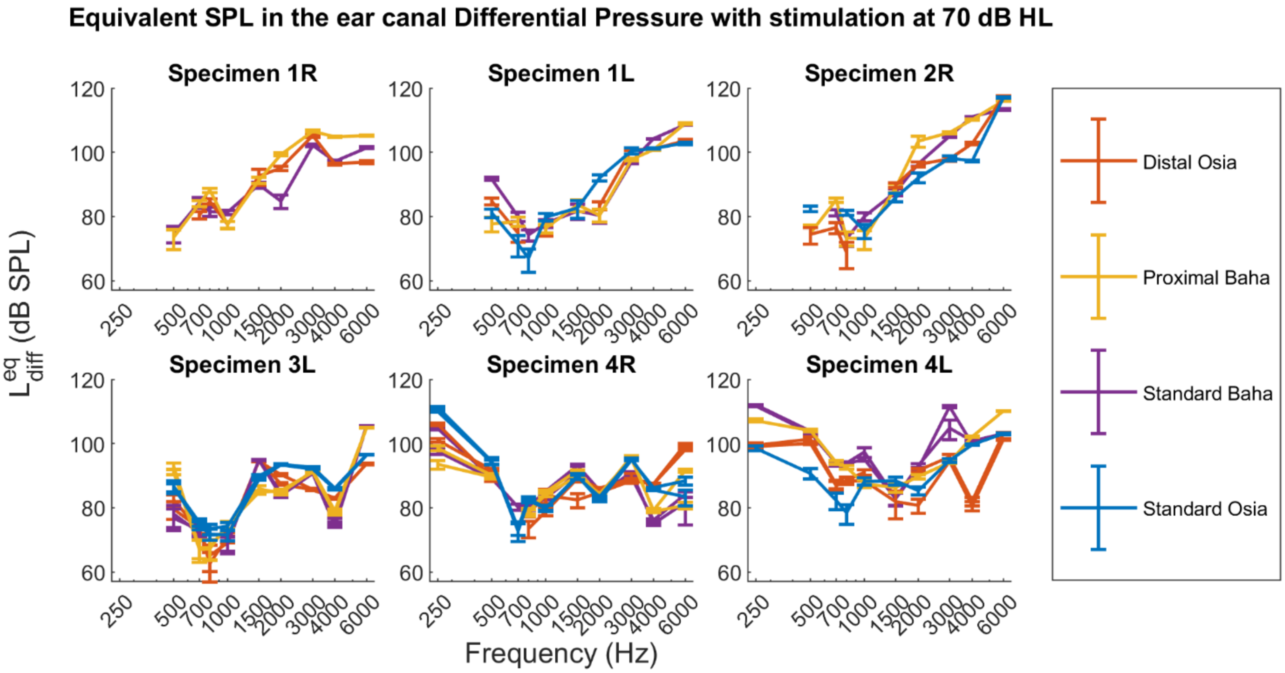

Supplementary Figure 29. Equivalent SPL in the ear canal for differential pressure for bone conduction stimulation in all specimens with stimulation at 70 dB HL. For specimen 1R, stimulation at the Standard Osia position was not performed.

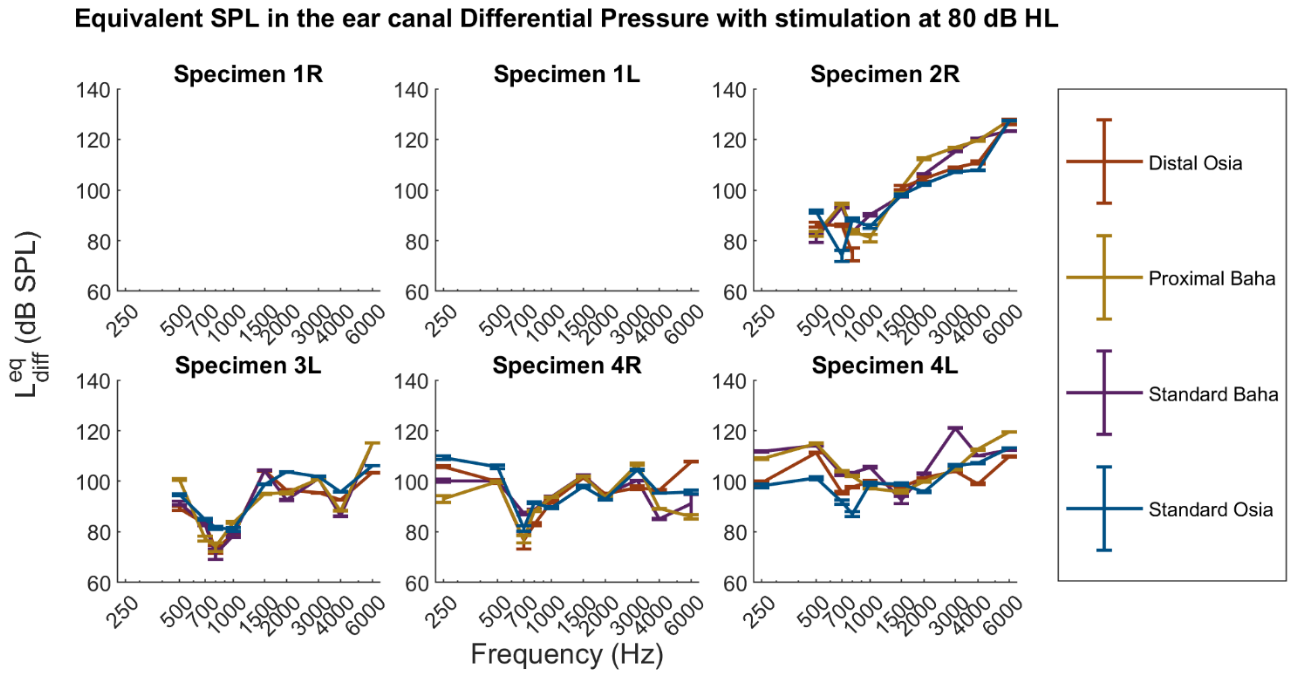

Supplementary Figure 30. Equivalent SPL in the ear canal for differential pressure for bone conduction stimulation in all specimens with stimulation at 80 dB HL. For specimens 1R and 1L, stimulation at 80 dB HL was not performed.

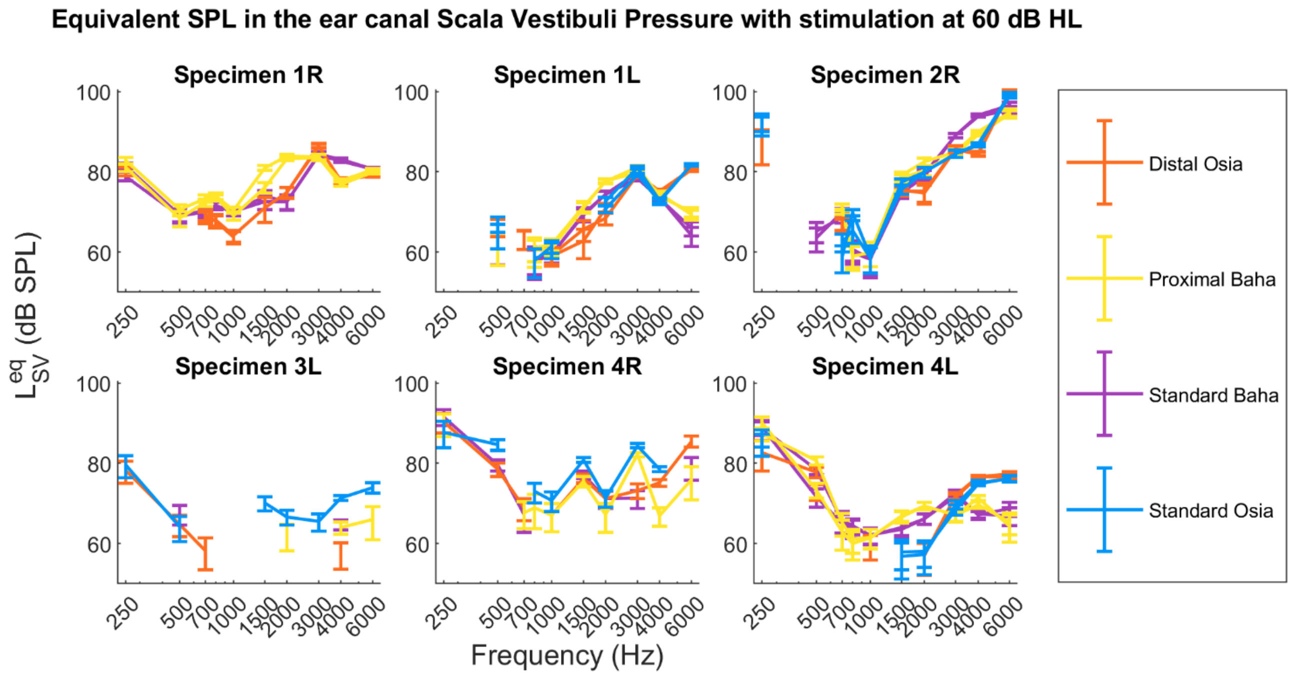

Supplementary Figure 31. Equivalent SPL in the ear canal for scala vestibuli pressure for bone conduction stimulation in all specimens with stimulation at 60 dB HL. For specimen 1R, stimulation at the Standard Osia position was not performed.

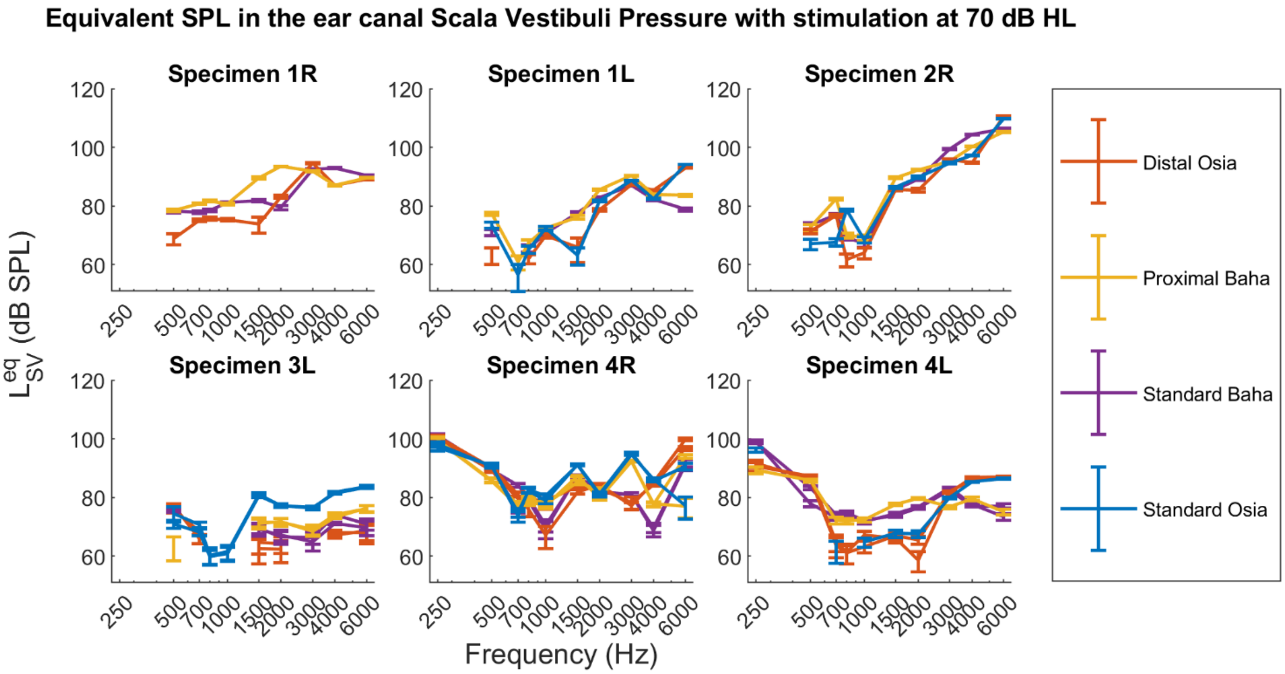

Supplementary Figure 32. Equivalent SPL in the ear canal for scala vestibuli pressure for bone conduction stimulation in all specimens with stimulation at 70 dB HL. For specimen 1R, stimulation at the Standard Osia position was not performed.

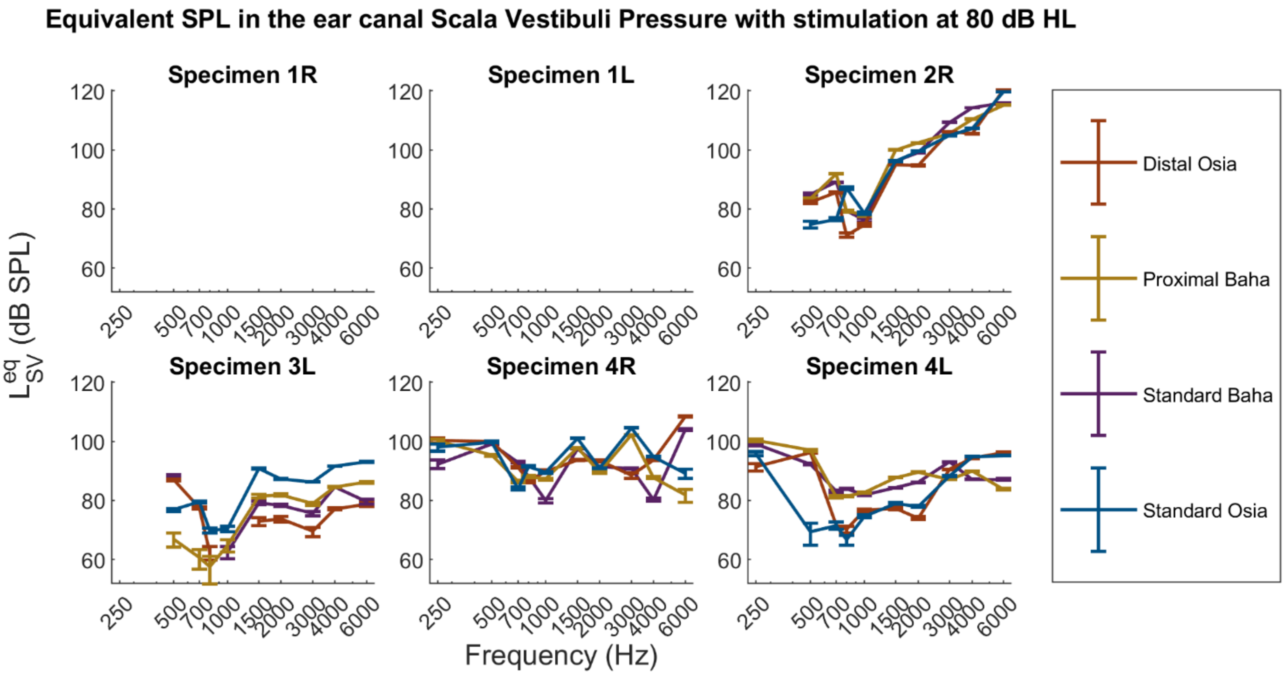

Supplementary Figure 33. Equivalent SPL in the ear canal for scala vestibuli pressure for bone conduction stimulation in all specimens with stimulation at 80 dB HL. For specimens 1R and 1L, stimulation at 80 dB HL was not performed.

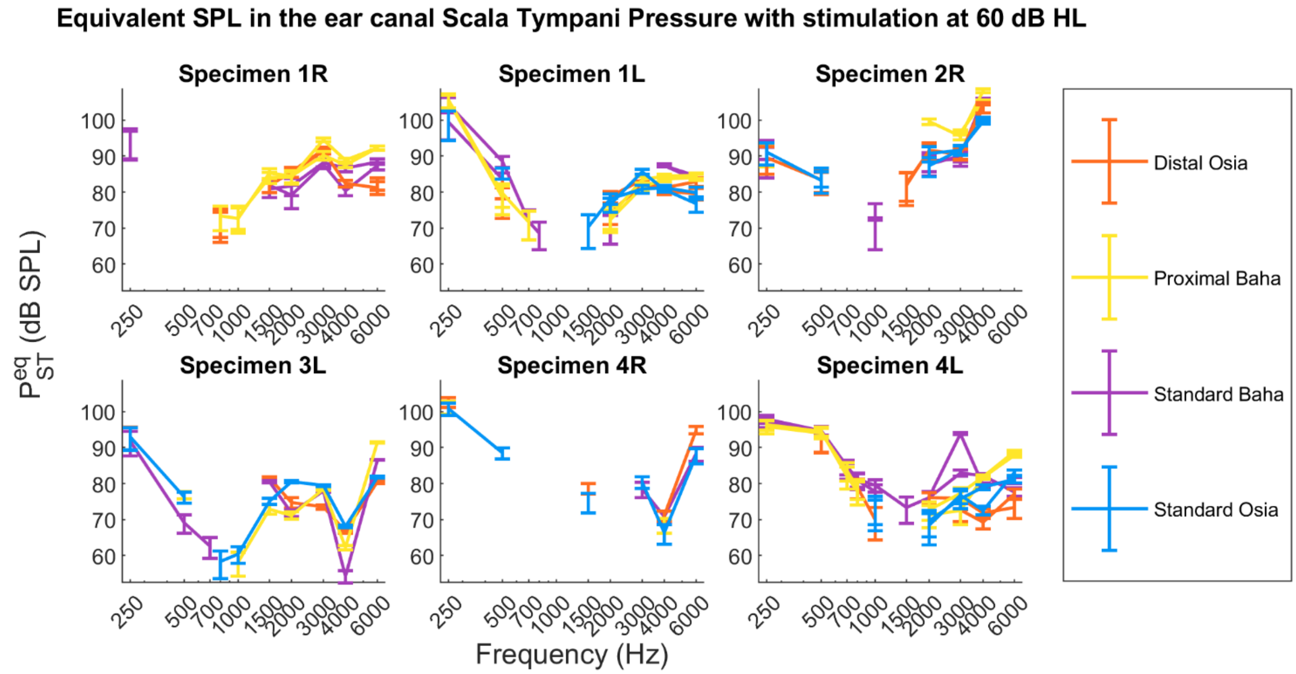

Supplementary Figure 34. Equivalent SPL in the ear canal for scala tympani pressure for bone conduction stimulation in all specimens with stimulation at 60 dB HL. For specimen 1R, stimulation at the Standard Osia position was not performed.

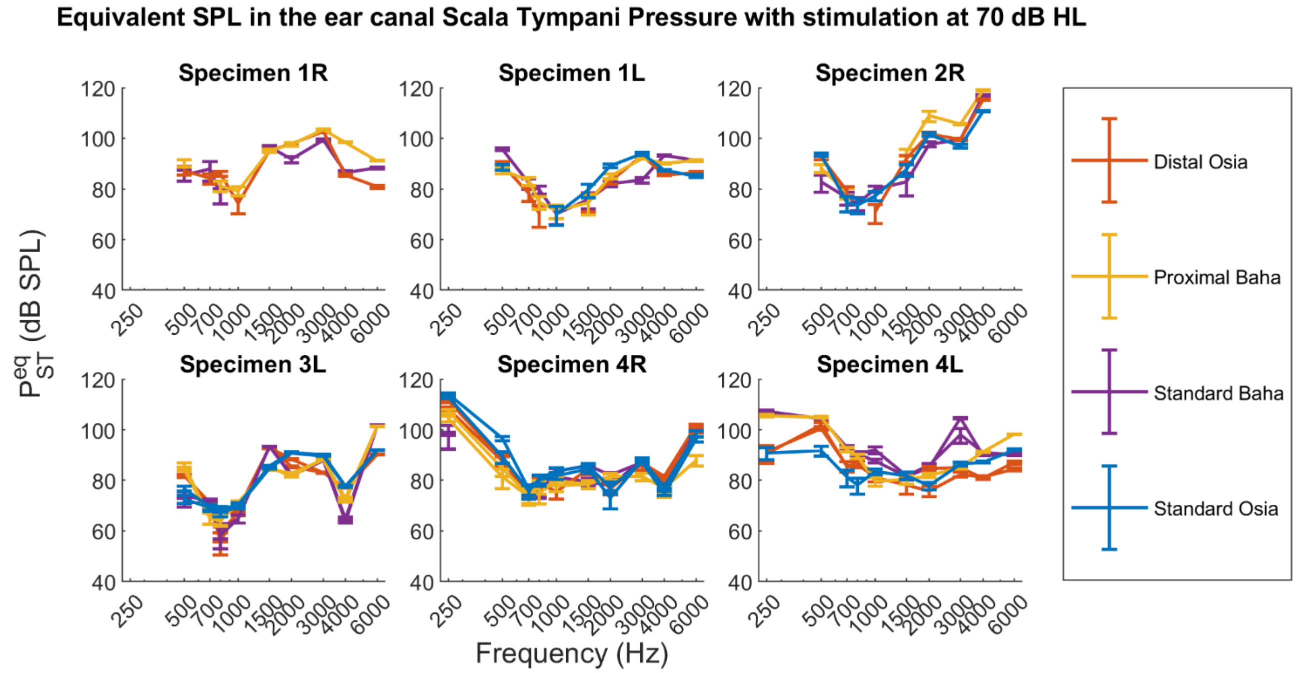

Supplementary Figure 35. Equivalent SPL in the ear canal for scala tympani pressure for bone conduction stimulation in all specimens with stimulation at 70 dB HL. For specimen 1R, stimulation at the Standard Osia position was not performed.

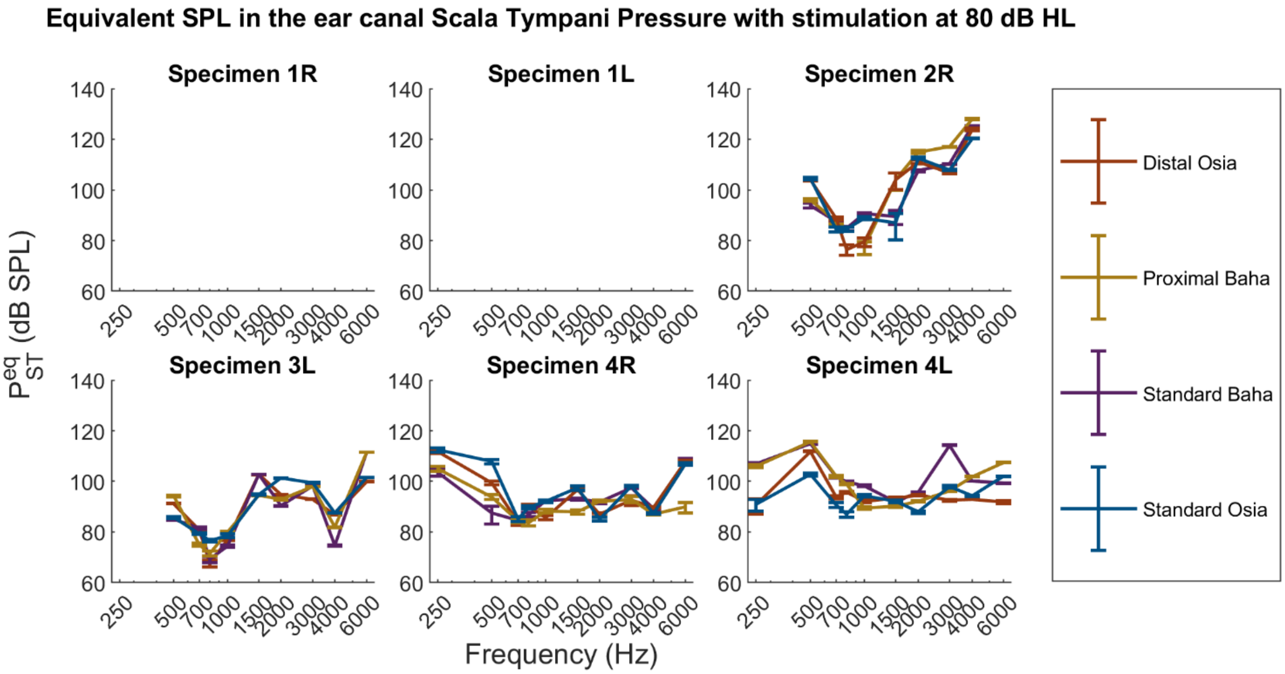

Supplementary Figure 36. Equivalent SPL in the ear canal for scala tympani pressure for bone conduction stimulation in all specimens with stimulation at 80 dB HL. For specimens 1R and 1L, stimulation at 80 dB HL was not performed.

6 Results for bone conduction: phase

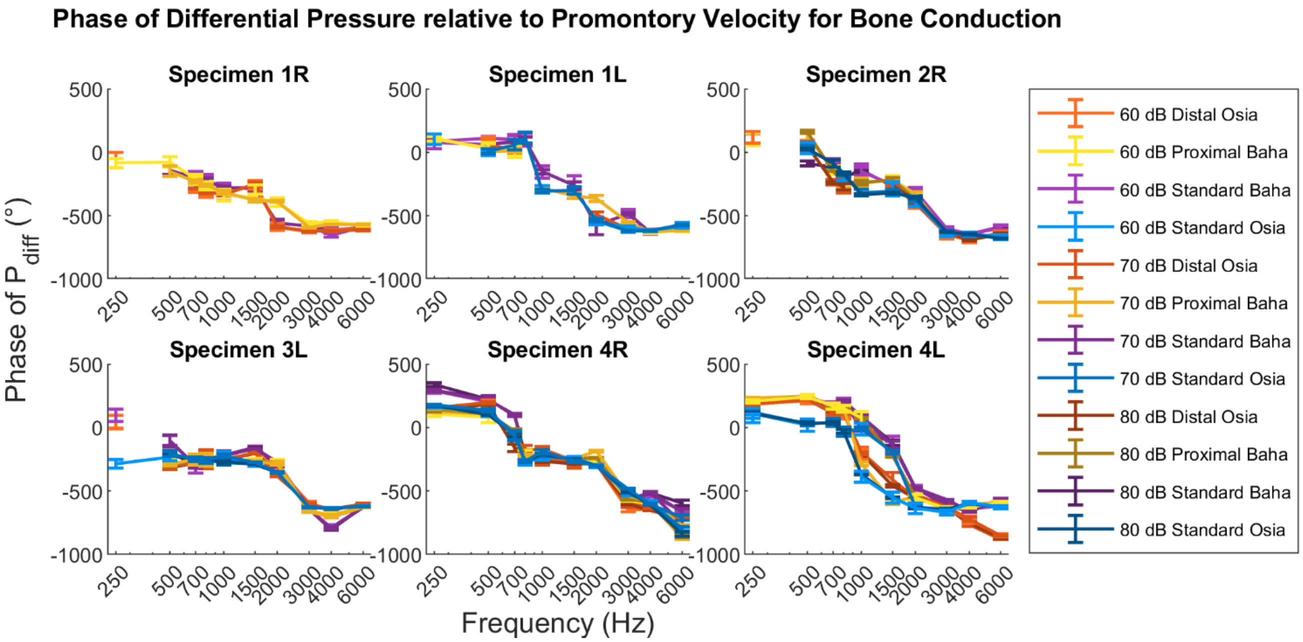

Supplementary Figure 37. The phase of the differential pressure for bone conduction stimulation in all specimens relative to the promontory velocity. For specimen 1R, stimulation at 80 dB HL and the Standard Osia position were not performed. For specimen 1L, stimulation at 80 dB HL was not performed.

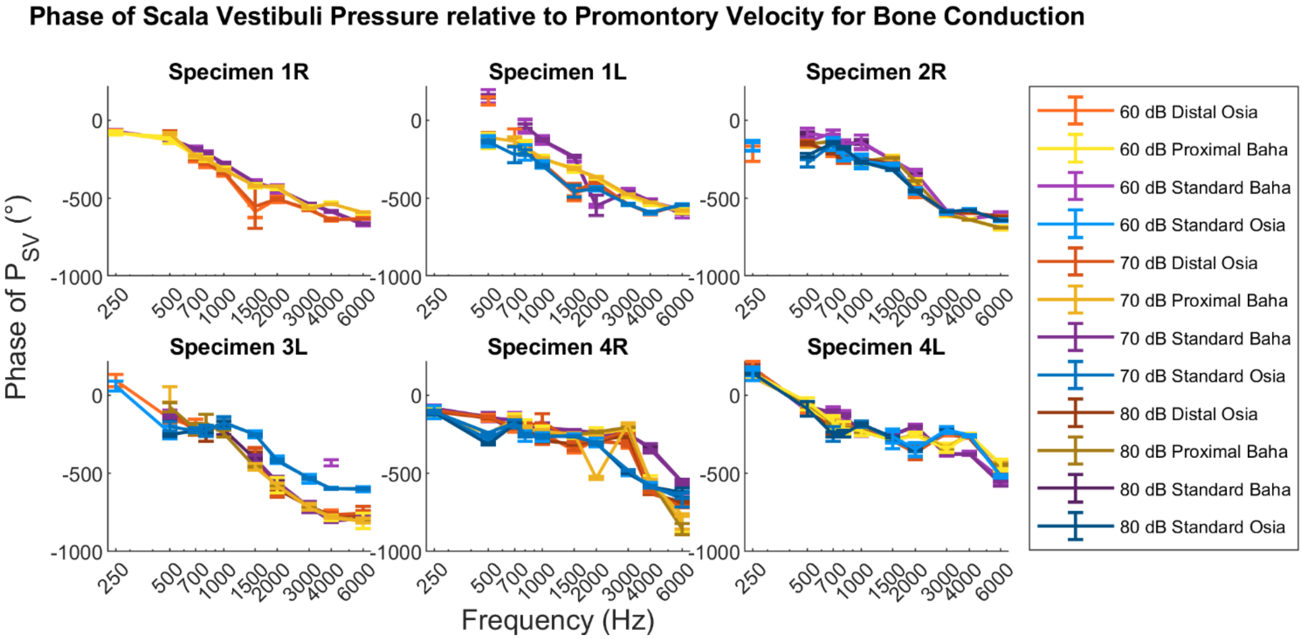

Supplementary Figure 38. The phase of the scala vestibuli pressure for bone conduction stimulation in all specimens relative to the promontory velocity. For specimen 1R, stimulation at 80 dB HL and the Standard Osia position were not performed. For specimen 1L, stimulation at 80 dB HL was not performed.

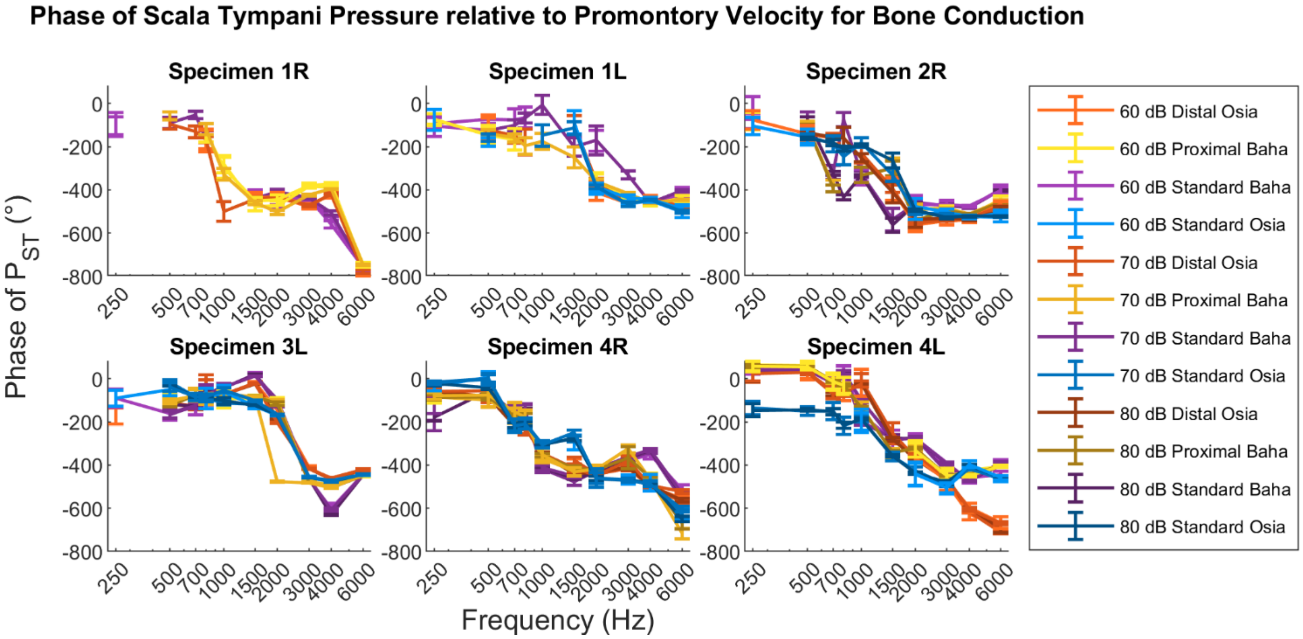

Supplementary Figure 39. The phase of the scala tympani pressure for bone conduction stimulation in all specimens relative to the promontory velocity. For specimen 1R, stimulation at 80 dB HL and the Standard Osia position were not performed. For specimen 1L, stimulation at 80 dB HL was not performed.

7 The most efficient stimulation position

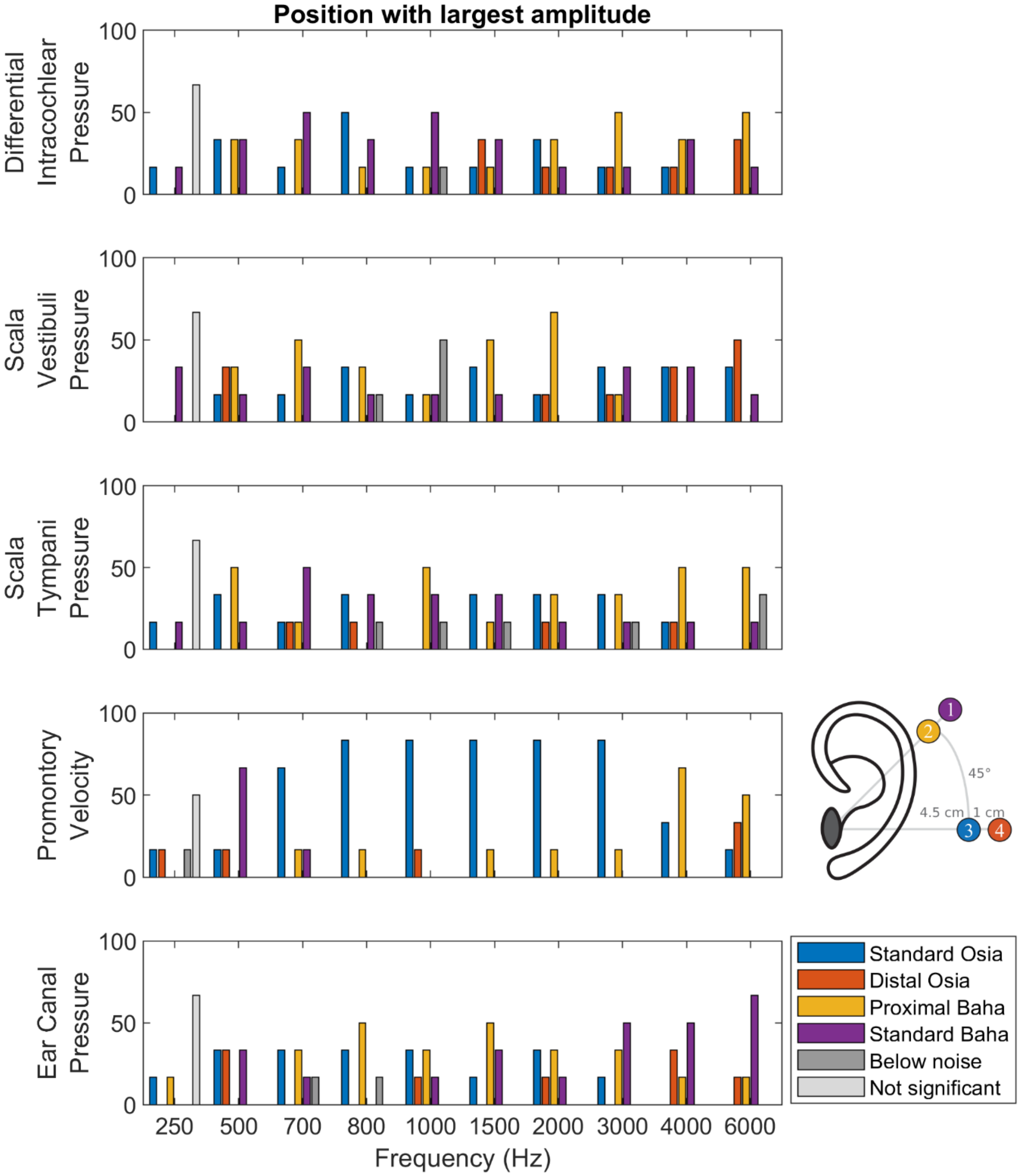

Supplementary Figure 40. The number of measurements (percentage representation) across specimens for which each stimulation position generated the highest amplitude per measurement technique and stimulation frequency.

8 Test-Retest variability

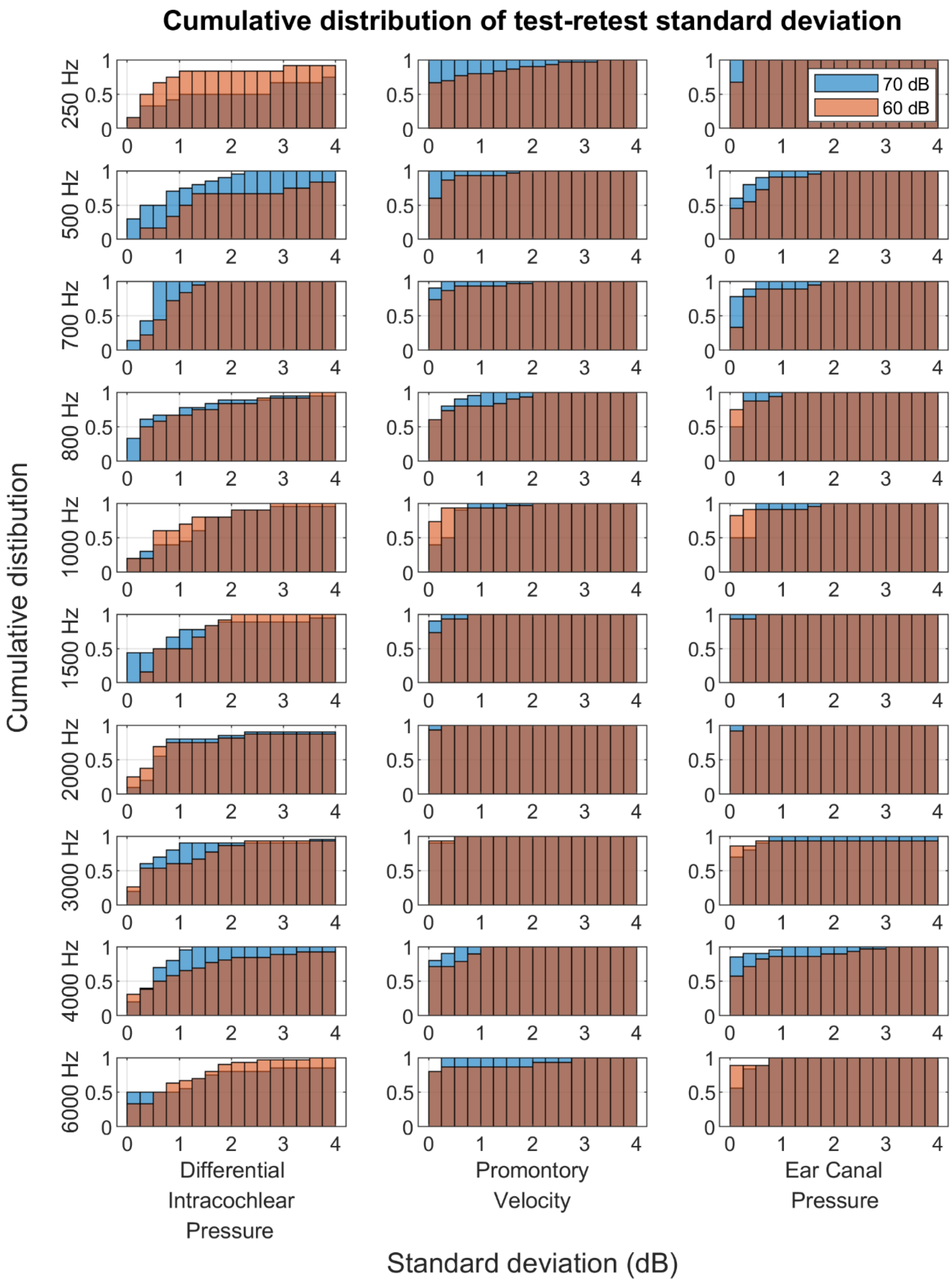

Supplementary Figure 41. Cumulative distribution of the test-retest variability.
